# Biodegradable poly(ε-caprolactone)/poly(silyl fumarate) shape memory scaffolds

**DOI:** 10.1016/j.polymer.2026.129694

**Published:** 2026-02-10

**Authors:** Jenlyan Negrón Hernández, Kaley Beach, Paola Chavarria, Melissa A. Grunlan

**Affiliations:** a Department of Chemistry, Texas A&M University, College Station, TX, 77843, United States; b Department of Materials Science and Engineering, Texas A&M University, College Station, TX, 77843, United States; c Department of Biomedical Engineering, Texas A&M University, College Station, TX, 77843, United States

**Keywords:** Poly(silyl fumarate), Caprolactone, Shape memory polymer

## Abstract

Biodegradable shape memory scaffolds have the unique potential to heal irregularly shaped craniomaxillofacial (CMF) defects through conformal ‘self-fitting’. These have been previously prepared from poly(ε-caprolactone) diacrylate (PCL-DA), but the slow biodegradation rate of PCL is expected to limit neotissue formation. Subsequently, telechelic siloxane macromers polydimethylsiloxane-dimethacrylate (PDMS-DMA) and polymethylhydrosiloxane-DMA (PMHS-DMA) were combined with PCL-DA at varying weight (wt)% ratios, resulting in PCL/PDMS and PCL/PMHS co-network scaffolds with accelerated degradation rates owing to phase separation that increased water uptake. Still, these siloxane macromers lack a hydrolytically unstable backbone thus limiting degradation. Herein, poly(silyl fumarate) (PSF) was synthesized as a hybrid siloxane macromer with a hydrolytically unstable backbone as well as interchain crosslinkability. PCL/PSF scaffolds were prepared at 90:10, 75:25, 60:40, and 50:50 wt% of PCL-DA to PSF via solvent-casting particulate leaching (SCPL) with a fused salt template. Despite a reduction in PCL crystallinity (i.e., switching segments) with 40 and 50 wt% PSF, all scaffolds maintained excellent shape memory behavior. PCL/PSF scaffolds with 10 and 25 wt% PSF also maintained the modulus of the PCL-only scaffold as well as the corresponding PCL/PDMS and PCL/PMHS scaffolds. *In vitro* degradation under basic conditions revealed that PCL/PSF scaffolds with just 10 wt% PSF degraded faster than the PCL-only scaffold and further increased with 25 wt% PSF to surpass that of the corresponding PCL/PDMS scaffold. A lack of phases separation was observed, and thus indicated that faster degradation was achieved by the hydrolytic instability of the PSF.

## 1. Introduction

Treatment of irregular shaped craniomaxillofacial (CMF) bone injuries with biological grafts is compromised by the inability to achieve a conformal fit and sufficient graft-to-tissue contact, leading to premature graft resorption [1–3]. Representing a regenerative engineering approach using off-the-shelf surgical product, we have previously reported “self-fitting” shape memory polymer (SMP) scaffolds based on biodegradable poly(ε-caprolactone) (PCL) [4,5]. Macroporous scaffolds (*d* ~220 μm, ~70% porosity) were fabricated from UV-curable PCL_90_-diacrylate (PCL_90_-DA, *M*_*n*_ ~10 kg mol^−1^) using a solvent-casting particular leaching (SCPL) protocol with a fused salt template. During conformal fitting into an irregular defect geometry, the netpoints are the covalent crosslinks while the switching segments are the crystalline lamellae (*T*_*m, PCL*_ or “*T*_*tran*s_” ~55 °C). After submersion in warm saline (*T* > *T*_*m, PCL*_), the lamellae melts, and the scaffold becomes malleable such that it can be press-fit into the defect while shape recovery prompts it to expand to the perimeter. Subsequent cooling to body temperature (*T* < *T*_*m, PCL*_) restores the scaffold to a relatively rigid state. Mechanical properties were characterized by non-brittle behavior (e.g., no fracture at 85% strain), and a modulus in the range of trabecular bone. The properties of PCL_90_-DA scaffolds were also not impacted by ethylene oxide (EtO) [6] and electron beam (E-beam) [7] sterilization. Additionally, PCL scaffolds fitted into model defects and then seeded with human mesenchymal stem cells (h-MSCs) exhibited osteogenesis [8]. *In vivo* studies further demonstrated that these scaffolds, owing to their good contact with adjacent tissue, supported bone tissue healing [9,10].

PCL is known to undergo slow degradation (~2 years) *in vivo* [11, 12]. Thus, while PCL SMP scaffolds display excellent properties, increasing the rate of biodegradation is critical to promote neotissue infiltration and bone defect healing. Crosslinkable PCL_90_-DA and thermoplastic poly(l-lactic acid) (PLLA) (*M*_*n*_ ~15 kg mol^−1^) were combined to form PCL/PLLA semi-interpenetrating network (semi-IPN) scaffolds with accelerated degradation due to phase separation effect [8,13]. SMP scaffolds may also be prepared by modification of PCL with siloxane polymers having *T*_*m*_s and/or glass transition temperatures (*T*_*g*_s) that are lower than that of PCL (*T*_*m*_ ~55 °C*, T*_*g*_ ~ −60 °C), such as polydimethylsiloxane (PDMS, *T*_*m*_ ~ −55 °C*, T*_*g*_ ~ −120 °C) [14]. We have previously reported SMP scaffolds formed as co-networks by combining a telechelic siloxane macromer with the PCL_90_-DA at varying ratios. Utilizing PDMS_66_-dimethacrylate (PDMS_66_-DMA) (*T*_*g*_ ~ −120 °C), co-network PCL/PDMS scaffolds were formed, exhibiting faster rates of degradation, likewise due to phase separation [15]. The use of relatively more hydrophilic polymethylhydrosiloxane-DMA (PMHS_60_-DMA) (*T*_*g*_ ~ −135 °C) produced faster degrading co-network PCL/PMHS scaffolds [16]. Both PCL/PDMS and PCL/PMHS scaffolds exhibited bioactivity, with hydroxyapatite (HAp) mineralization (simulated body fluid, SBF 1X) occurring at 4 and 2 weeks, respectively. Additionally, shape memory behavior was maintained owing to the retention of PCL % crystallinity as for the PCL-only scaffold. Yet, scaffold degradation remains limited by the hydrolytically stability of the Si–O–Si polymeric backbone for such telechelic siloxane macromers [17]. Additionally, the exceptionally low *T*_*g*_s of the telechelic siloxane macromers impart substantial reductions in scaffold strength as well as modulus depending on concentration.

**Fig. 1.**
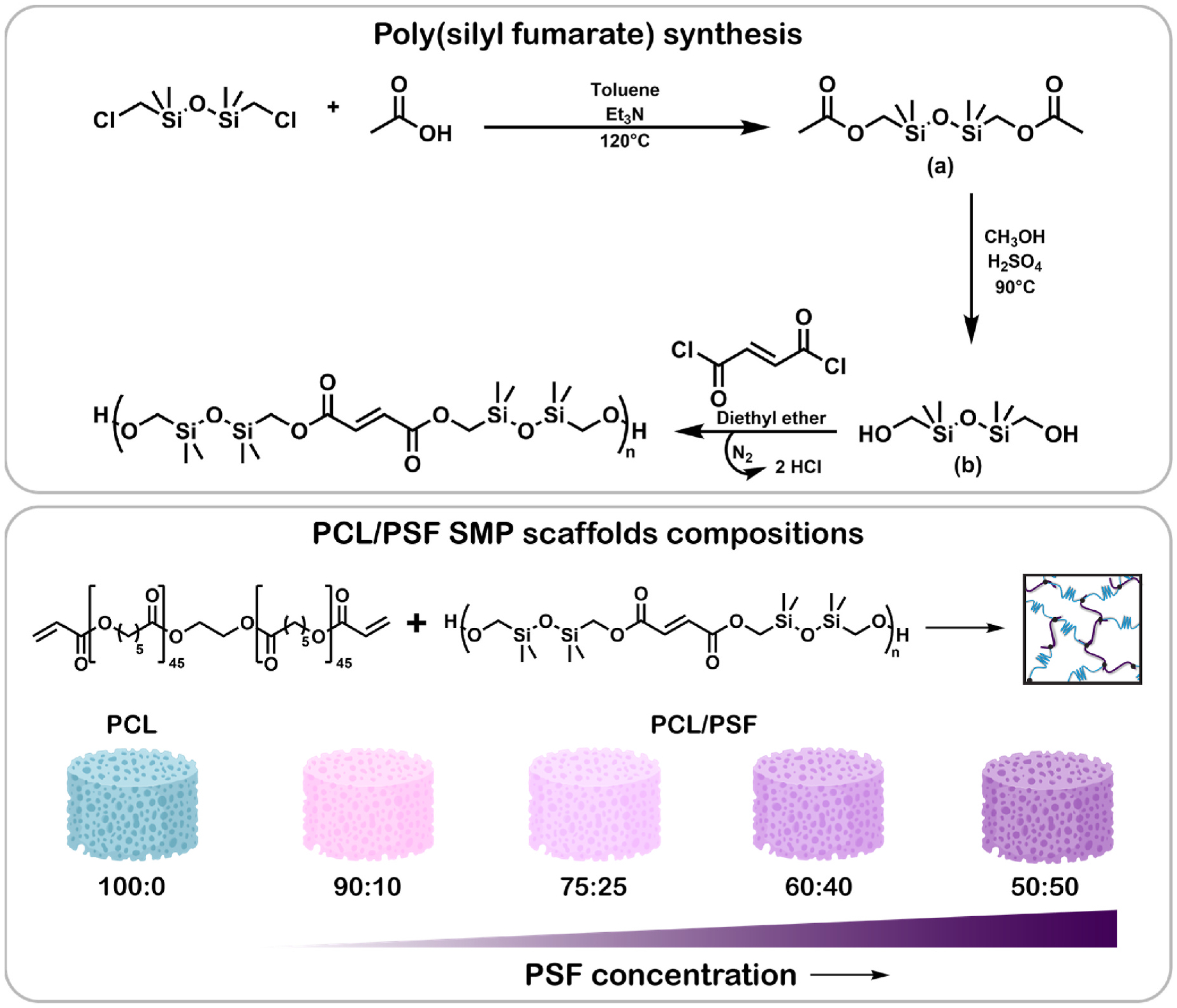
**(top)** Poly(silyl fumarate) (PSF) synthesis scheme. **(bottom)** Combination of poly(ε-caprolactone)_90_-diacrylate (PCL_90_-DA) and PSF at varying wt% ratios to form co-networks as shape memory scaffolds.

Herein, a new siloxane-containing macromer, poly(silyl fumarate) (PSF), was synthesized and combined with PCL_90_-DA to form PCL/PSF SMP scaffolds (Fig. 1). Poly(propylene fumarate) (PPF) has been extensively used for bone tissue regeneration [18–23], as well as for other medical applications (e.g., adhesives [24–26], drug delivery [27–30]). PPF is amorphous and the *T*_*g*_ (~−30 °C to ~30 °C) varies with molecular weight [31]. Unsaturated carbon-carbon (C=C) moieties of the PPF backbone afford crosslinking among PPF chains or added crosslinkers. Following hydrolysis of its ester linkages, PPF degradation byproducts can be readily excreted by the body [32–35]. Multi-block copolymers of PPF and PCL (i.e., PPF-*co*-PCL) have been synthesized and used to fabricate porous scaffolds via thermally-induced phase separation [22,36] and also evaluated as an injectable scaffold for vertebroplasty [37]. Creating macroporous SMP scaffolds using a SCPL protocol [4,5] is limited by the immiscibility between PCL_90_-DA and PPF when combined in DCM to cast over the fused salt template [38]. Thus, a ‘hybrid’ PSF was prepared to introduce siloxane moieties, affording a macromer with a hydrolytically unstable backbone and interchain crosslinkability, as well as amenable to SCPL fabrication of SMP scaffolds in combination with PCL_90_-DA. In this study, the PSF macromer was synthesized via step-growth polymerization of fumaryl chloride and 1,3-Bis(hydroxymethyl)tetramethyldisiloxane. PCL/PSF co-network solid films were initially formed with 10 to 90 wt% PSF to assess impact on crosslinking as well as thermal and shape memory behaviors. Based on these studies, porous scaffolds were subsequently fabricated with up to 50 wt% PSF. The salt size of the fused salt template was adjusted based on PSF content to afford similar pore size. The PCL/PSF co-network scaffolds were assessed in terms of thermal, shape memory, mechanical and degradation properties as well as bioactivity, and compared to that of analogous PCL/PDMS and PCL/PMHS scaffolds.

## 2. Materials and methods

### 2.1. Materials

Poly(ε-caprolactone)-diol [PCL_90_-diol, *M*_*n*_ ~10 kg mol^−1^], triethylamine ≥99% (Et_3_N), acryloyl chloride [≥97.0%, contains ~400 ppm of phenothiazine as stabilizer], 4-dimethylaminopyridine [ReagentPlus^®^, ≥99%] (DMAP), 2,2-dimethoxy-2-phenylacetophenone 99% (DMP), 1-vinyl-2-pyrrolidone ≥99% (NVP), potassium carbonate [ACS reagent, ≥99.0%] (K_2_CO_3_), sodium hydroxide ≥97% (NaOH), potassium hydroxide [ACS reagent, ≥85%] (KOH), sodium chloride [ACS reagent, ≥99.0%] (NaCl), anhydrous magnesium sulfate ≥99.5%] (MgSO_4_), glacial acetic acid [ACS reagent, ≥99.7%], diethyl ether [ACS reagent, ≥99.0%], methanol certified ACS, sulfuric acid 95.0 – 98.0% (H_2_SO_4_), fumaryl chloride 95%, and NMR-grade CDCl_3_ were obtained from Sigma-Aldrich. 1,3-Bis(chloromethyl)tetramethyldisiloxane 95% was obtained from Gelest. Reagent-grade dichloromethane (DCM), ethyl acetate (EtOAc), and toluene were dried over 4 Å molecular sieves prior to use.

### 2.2. Syntheses

Monomers and polymers were synthesized under a nitrogen (N_2_) atmosphere with a Teflon-covered stir bar to agitate the mixture. Product structures were determined via ^1^H NMR spectroscopy on a Bruker Avance Neo 400 MHz spectrometer operating in FT mode with CDCl_3_ as the standard. Size-exclusion chromatography (SEC) analysis was performed on a TOSOH Ambient Temperature SEC system equipped with a refractive index (RI) detector. TSKgel SuperHZ4000 (150 mm × 4.6 mm) column packed with highly crosslinked polystyrene/divinylbenzene copolymer was maintained at 30 °C in a column oven. The eluting solvent was HPLC grade dimethylformamide (DMF) at a flow rate of 0.16 mL/min. The detector was calibrated with a polystyrene standard with the following *M*_*w*_ (g mol^−1^) fractions: 589; 1010; 3120; 5430; 13,700; 37,200; 18,9000; and 39,7000. Data analysis was performed with EcoSec Analysis.

#### 1,3-Bis(acetoxymethyl)tetramethyldisiloxane.

1,3-Bis(acetoxymethyl) tetramethyldisiloxane **(a)** was prepared as reported [39] with modifications (Fig. 1). 1,3-Bis(chloromethyl)tetramethyldisiloxane (7.0 g, 30 mmol) was dissolved in toluene (120 mL), and reacted with acetic acid (4.19 g, 70 mmol) in the presence of Et_3_N (7.70 g, 76 mmol). The reaction was refluxed at 120 °C for 72 h. Next, the triethylamine hydrochloride salts were removed by gravity filtering. Toluene was removed via rotary evaporation, the resulting crude product was dissolved in diethyl ether (50 mL), and washed with 10 mL of deionized (DI) water in a separatory funnel. After overnight (O/N) separation, the organic layer was collected, dried with MgSO_4_, gravity filtered, solvent removed via rotary evaporation, and dried under vacuum to obtain purified **a** [~5.0 g, 71% yield]. ^1^H NMR agreed with that previously reported (Fig. S1).

#### 1,3-Bis(hydroxymethyl)tetramethyldisiloxane.

1,3-Bis(hydroxymethyl) tetramethyldisiloxane **(b)** was prepared as reported [39] with modifications (Fig. 1). 1,3-Bis(acetoxymethyl)tetramethyl-disiloxane **(a)** (5.0 g) was dissolved in excess methanol (75 mL) with sulfuric acid as a catalyst. The reaction was refluxed at 90 °C for 20 h. Next, the acid was neutralized by adding a pellet of KOH. Using rotary evaporation, methanol was removed and the resulting crude product dissolved in diethyl ether (50 mL) and washed with 10 mL of DI water in a separatory funnel. After O/N separation, the organic layer was collected, dried with MgSO_4_, gravity filtered, solvent removed via rotary evaporation, and the liquid dried under vacuum to obtain purified **b** [~3.5 g, ~70% yield]. ^1^H NMR agreed with that previously reported (Fig. S2).

#### PSF.

PSF (*M*_*w*_ ~16 kg mol^−1^) was prepared via a protocol adapted from Hedberg-Dirk et al. (Fig. 1) [3,40]. 1,3-Bis(hydroxymethyl)tetramethyldisiloxane **(b)** (7.0 g, 36 mmol) was dissolved in diethyl ether (50 mL) in a three-neck round bottom (rb) flask set in an ice bath. The solution was sparged with N_2_ for 30 min through an adapter fitted with a fine-fritted gas dispersion tip and forced via a plastic tube into a secondary vessel containing a 1 M NaOH solution. Next, fumaryl chloride (4.58 g, 30 mmol) was dissolved in diethyl ether (7 mL) and added dropwise to the 1,3-Bis(hydroxymethyl)tetramethyldisiloxane solution while stirring. Additional diethyl ether was subsequently added to the reaction mixture to bring the volume back to 50 mL. The reaction was allowed to stir O/N with sparging. Diethyl ether was carefully removed via rotary evaporation, and the resulting crude product dissolved in DCM (50 mL), and washed with 10 mL of DI water in a separatory funnel. After O/N separation, the organic layer was collected, dried with MgSO_4_, gravity filtered, the solvent removed via rotary evaporation, and the resulting purified PSF [~4.6 g, ~65% yield] dried under vacuum. The molecular structure was confirmed via ^1^H NMR spectroscopy (Fig. S3). The SEC trace revealed 3 peaks, weight average molecular weight (*M*_*w*_), number average molecular weight (*M*_*n*_), and dispersity (*Đ*) values were obtained as, respectively: ~95 kg mol^−1^, ~83 kg mol^−1^, 1.2 [peak 1]; ~9 kg mol^−1^, ~5 kg mol^−1^, 1.8 [peak 2]; and ~300 g mol^−1^, ~100 g mol^−1^, 1.8 [peak 3] (Table S1, Fig. S4). Overall, SEC analysis determined a *M*_*w*_ of ~16 kg mol^−1^ for an average degree of polymerization (*n*) of 60. The DSC thermogram of PSF revealed a single endothermic *T*_*g, PSF*_ peak at ~ −67 °C (Table S2, Fig. S5). TGA analysis (RT to 500 or 600 °C, at a heating rate of 10 °C min^−1^) revealed a single onset of catastrophic degradation at ~320 °C (Fig. S6a).

#### PCL_90_-DA.

PCL_90_-DA (*M*_*n*_ ~10 kg mol^−1^) was prepared by acrylation of PCL_90_-diol (*M*_*n*_ ~10 kg mol^−1^) as previously reported [5]. PCL_90_-diol (20.0 g, 2.0 mmol) and DMAP (6.6 mg, catalyst) were dissolved in dry DCM (120 mL). Et_3_N (0.56 mL, 4.0 mmol) and acryloyl chloride (0.65 mL, 8.0 mmol) were added sequentially and dropwise to the flask via a needle and syringe through a rubber septum. The reaction was stirred at room temperature (RT) for 30 min and subsequently refluxed at 55 °C for 20 h. DCM was removed via rotary evaporation, and the crude was dissolved in dry EtOAc (135 mL), and gravity filtered to remove triethylamine hydrochloride salts. After removing the solvent, the product was dissolved in DCM (140 mL) and washed with 2 M K_2_CO_3_ (13.5 mL). After O/N separation, the organic layer was collected, dried with MgSO_4_, gravity filtered, solvent removed via rotary evaporation, and dried under vacuum to obtain PCL_90_-DA (~80% yield). ^1^H NMR (Fig. S7) and DSC agreed with that previously reported. *M*_*n*_ ~10 kg mol^−1^; >90% acrylation; *T*_*m, PCL*_ = ~53 °C; PCL % crystallinity = ~48%.

### 2.3. Film fabrication

Solid films were formed via solvent casting with varying wt% ratios of PCL_90_-DA and PSF (100:0, 90:10, 75:25, 60:40, 50:50, 40:60, 25:75, 10:90, and 0:100). Precursor solutions were prepared by dissolving their respective macromer(s) (1.0 g macromer total in 2.26 mL DCM) and 15 vol% of a photoinitiator solution (10 wt% DMP in NVP) atop a shaker plate in a closed vial. The precursor solutions were pipetted into individual circular silicone molds (*d* ~45 mm × *t* ~2 mm, McMaster-Carr) placed between two glass slides, and exposed to UV light (UV-Transilluminator, 6 mW cm^2^, 365 nm) for 4 min (2 min per side). The resulting solvent-swollen discs were air dried (RT, 12 h), removed from the molds carefully, and dried *in vacuo* (RT, 4 h, 30 in. Hg). Uncrosslinked material was removed by soaking the discs in ethanol (3 h). The discs were then sequentially air-dried (RT, 12 h) and annealed *in vacuo* (85 °C, 1 h, 30 in. Hg). After 48 h at RT, specimens were biopsy punched (Integra Miltex, *d* ~6 mm). Final dimensions of film disc specimens were *d* ~6 mm × *t* ~1.1 mm.

### 2.4. Scaffold fabrication

Scaffolds with interconnect macropores were fabricated via a SCPL method with a fused salt template, as previously reported [41]. NaCl was sieved (sieves with 500, 425, 355, and 250 μm openings) to obtain a size of 449 ± 36 μm, 380 ± 58 μm, or 285 ± 70 μm, respectively. Scanning electron microscopy (SEM; JEOL 6400 SEM, 10 kV accelerating voltage) was used in conjunction with imaging software ImageJ^®^ to determine the average salt size. A designated sieved salt (10 g) was placed in a 20 mL glass scintillation vial (I.D. = 25 mm), to which DI water was added in four portions and stirred with a spatula. The hydrated salt was compacted to the bottom of the vial using a blunt glass rod, and the capped vial was then centrifuged (3220 × *rpm*, 15 min). The vials were subsequently opened to air-dry (RT, 1 h), then dried *in vacuo* (RT, 12 h, 30 in. Hg), resulting in a fused salt template.

Scaffolds were formed with varying wt% ratios of PCL_90_-DA and PSF (100:0, 90:10, 75:25, 60:40, and 50:50), representing compositions with adequate shape memory behavior as films. Precursor solutions were prepared with their respective macromer(s) (0.15 g per mL DCM) and 15 vol% of a photoinitiator solution (10 wt% DMP in NVP). The resulting precursor solution (~5 mL) was added to a fused salt template. The vials were centrifuged (1260 × *rpm*; 10 min), opened and exposed to UV light for 10 min [100:0 and 90:10 scaffolds; UV-Transilluminator (6 mW cm^−2^, 365 nm), and 75:25, 60:40, and 50:50; UV Cure Box IntellyRay 400 (115 mW cm^−2^, 365 nm)]. Following air-drying (RT, 12 h), the salt template was removed from the scaffolds by soaking in a water/ethanol solution (1:1 vol:vol) for ~5 days with daily solution changes and with scaffolds removed from vials after day 2. The resulting cylindrical scaffolds were allowed to air dry (RT, 12 h), annealed *in vacuo* (85 °C, 1 h, 30 in. Hg), and maintained at RT for 48 h. The cylindrical scaffolds (*d* ~12 mm) were then sliced (*t* ~2 mm) (Vibratome, Leica VT 1000 S), and subsequently biopsy punched (Integra Miltex, *d* ~6 mm). Final dimensions of scaffolds disc specimens were *d* ~6 mm × *t* ~2 mm.

### 2.5. Characterization of films and scaffolds

#### 2.5.1. Sol content

Solid films (*N* = 3) and porous scaffold (*N* = 3) specimen discs were weighed, and each submerged in 10 mL DCM within a 20 mL scintillation vial. Sealed vials were placed atop a shaker plate (48 h, 150 rpm). Specimens were sequentially removed from DCM, air dried (RT, O/N), dried *in vacuo* (RT, ON, 30 in. Hg) and weighed. The initial and final weights of specimens were used to determine % sol content.

#### 2.5.2. Thermal gravimetric analysis

TGA (TA Instruments Q50) was performed using solid films (*N* = 3) and porous scaffolds (reduced to a size of *d* ~4 mm × *t* ~2 mm) (*N* = 3) in platinum pans under N_2_ from RT to 500 or 600 °C, at a heating rate of 10 °C min^−1^.

#### 2.5.3. Porosity (%), pore size, and pore interconnectivity

Percent porosity of scaffold discs (*N* = 3) was determined by following equation (1):

$$\text{Porosity}(\%)=\frac{\rho_{\text{film}}-\rho_{\text{scaffold}}}{\rho_{\text{film}}}\times 100$$

Scaffold pore size and pore interconnectivity were evaluated via SEM. Scaffold cross-sections were subjected to Au–Pt coating (~4 nm). From the SEM images (Tescan Vega 3 SEM), the average pore size was determined from measurements (*N* = 30) of pores measured along each image midline with ImageJ software.

#### 2.5.4. PCL T_m_ and % crystallinity of films and scaffolds

PCL melting temperature (*T*_*m, PCL*_) and % crystallinity of solid films and scaffolds were determined via DSC (TA Instruments Q100). Specimens (*N* = 3) (~15 mg) were individually sealed in hermetic platinum pans and heated at a rate of 5 °C min^−1^ from RT to 100 °C. Values were collected from the 2nd DSC cycle to remove thermal history. The *T*_*m*_ was determined from the endothermic melt peak’s maximum value, and % crystallinity (% *X*_*c*_) was calculated with equation (2). Δ*H*_*m*_ is the enthalpy of fusion from the endothermic melt peak and $\Delta H_{c}^{\circ }$ is the enthalpy of fusion of theoretical 100 % crystalline PCL (139.5 J g^−1^). *w* is the mass fraction of the PCL (e.g., *w* = 0.90 for PCL and *w* = 0.10 for PSF in the case of PCL/PSF [90:10]).

$$\%X_{c}=\frac{\Delta H_{m}}{\Delta H_{c}^{\circ }\times w}\times 100$$

#### 2.5.5. Shape memory behavior

Qualitative shape memory behavior of flat, rectangular films (i.e., ‘permanent shape’) (*l* ~30 mm × *w* ~30 mm × *t* ~1 mm) were subjected to the following sequence: (1) after exposure to a water bath at 55 °C (*T*_*m, PCL*_) for 1 min, immediately deform into a coiled shape (i.e., ‘temporary shape’) by tightly wrapping around a metal mandrel (*d* ~3 mm), (2) secure the specimen on the mandrel for ~2 min to allow for shape fixity; (3) remove the specimen from the mandrel and observe shape fixity at t = ~2 min, and (4) return the shape fixed specimen to the 55 °C water bath and observe shape recovery at t = 8 s.

Shape memory properties of scaffold specimens (*N* = 3) were quantitatively evaluated using a model defect representative of a rat calvarial defect, as previously described [42–44]. A circular defect (*d* ~5 mm) was created from an UHMWPE sheet (*t* ~2 mm) using a drill press (Grizzly G7948). A hot plate equipped with a digital temperature probe (Heidolph, MR HEI-TEC) was used to pre-heat water to ~55 °C. Each scaffold specimen was subjected to the following protocol, and the diameter recorded as noted with electronic calipers (resolution 0.01 mm) to quantify scaffold strain (*ε*): (1) record initial diameter; (2) submerge into the water bath at the designated *T*_*m, PCL*_ for ~1 min; (3) remove from the water bath and immediately press fit into the model defect at RT; (4) maintain in the defect for ~2 min to allow for shape fixity; (5) remove from the defect, and allowed to sit for ~2 min at RT; (6) record diameter; (7) re-submerge into the water bath at designated *T*_*m, PCL*_ for ~1 min to allow for shape recovery; (8) remove from the water bath and cool for ~2 min at RT; and (9) record diameter. This procedure was repeated twice to determine shape fixity (*R*_*f*_) and shape recovery (*R*_*r*_) over for the first (*C* = 0) and second (*C* = 1) cycle according to their respective equations.

$$R_{f}(N)=\frac{\varepsilon_{u}(N)}{\varepsilon_{m}}\times 100$$

$$R_{r}(N)=\frac{\varepsilon_{i}(N)}{\varepsilon_{r}(N)}\times 100$$

where ε_u_(*N*) is the scaffold diameter after press-fitted into the mold, *ε*_*m*_ is the diameter of the mold, *ε*_*i*_*(N)* is the scaffold diameter after shape-recovery, and *ε*_*r*_*(N)* is the initial diameter of the scaffold.

#### 2.5.6. Compressive mechanical properties

Scaffold discs (*N* = 5) underwent static compression tests (Instron 5944) at RT, and constant strain rate (1.5 mm min^−1^) up to 85% strain. Modulus (*E*) was determined from the resulting stress versus strain curves, specifically from the slope in the initial linear region (≤ 10% *ε*). Compressive strength (*CS*) was determined from the stress at 85% strain.

#### 2.5.7. Degradation

The degradation of scaffolds was conducted under accelerated, basic conditions (0.2 M NaOH) in accordance with ASTM F1635. Scaffold discs (*N* = 3 per time point) were each submerged in 10 mL of the solution in a sealed 20 mL glass scintillation vial and kept at 37 °C at 120 rpm (VWR Benchtop Shaking Incubator Model 1570). At each time point (1, 2, 3, 4, 5, 6 and 7 days), specimens were removed, rinsed with DI water, blotted, and dried *in vacuo* (RT, 12 h, 30 in. Hg). The % mass loss was determined by comparing the specimen’s original mass versus that of the specimen at the designated time point. Specimens were used for one time point (i.e., not used for subsequent time points).

#### 2.5.8. Bioactivity

A solution of simulated body fluid (SBF, 1X) was prepared as described by *Kokubu* et al. [45] Scaffold discs (*N* = 3) were placed in a sealed centrifuge tube containing ~10 mL SBF and submerged in a water bath at 37 °C. At 1-week, 2-week, and 4-week time points, scaffolds were removed from the solution, rinsed with DI water, and dried under vacuum (RT, O/N, 30 in. Hg). Specimens were coated with Au–Pt (~10 nm) and analyzed using SEM to visualize HAp mineralization on the pore walls.

### 2.6. Statistical analyses

Data was reported as a mean ± standard deviation. Values were compared in GraphPad Prism via ANOVA tests followed by Tukey’s method to test all pairwise mean comparisons and statistical significance was set to be at a *p-value <0.05.

## 3. Results and discussion

### 3.1. PSF syntheses

^1^H NMR confirmed the synthesis of 1,3-Bis(acetoxymethyl)tetramethyldisiloxane **(a)** (Fig. S1), and 1,3-Bis(hydroxymethyl)tetramethyldisiloxane **(b)** (Fig. S2). PSF was subsequently formed via step-growth polymerization of **(b)** and fumaryl chloride, and its molecular structure verified by ^1^H NMR (Fig. S3). A *M*_*w*_ of ~16 kg mol^−1^ (Đ ~28) was determined via SEC (Table S1, Fig. S4), for an average degree of polymerization (*n*) of ~60. DSC revealed that PSF was amorphous and displayed a *T*_*g*_ of −67 °C (Table S2, Fig. S5). A single *T*_*g*_ value is consistent with an alternating copolymer formed via step growth polymerization (i.e., comprised of alternating fumaric and siloxane units). Moreover, the −67 °C *T*_*g*_ value falls between that of PDMS (*T*_*g*_ ~ −120 °C) and PPF (*T*_*g*_ ~ −30 °C to ~30 °C, as *M*_*n*_ increases from ~11 to ~18 kg mol^−1^) [31].

### 3.2. Film fabrication

The UV cure of PCL_90_-DA and PSF, owing to their respective acrylate groups and unsaturated C=C bonds, was expected to give rise to co-networks. Prior to fabrication of scaffolds, films were prepared to verify co-network formation, among other properties as described below. PCL/PSF films and controls were prepared with tunable wt% ratios of PCL_90_-DA to PSF: 100:0 (‘PCL-only control’), 90:10, 75:25, 60:40, 50:50, 40:60, 25:75, 10:90, and 0:100 (‘PSF-only control’). The relatively lower onset of degradation temperature of PSF and greater char residue (Fig. S6a) was systematically reflected in the TGA thermograms of PCL/PSF co-network films of increasing PSF content (Fig. S6b). The successful formation of co-networks was also confirmed with sol content studies (Table S3). For PCL/PSF films up to 50 wt% PSF, sol content values (< ~7 %) were similar to that of the PCL-only (100:0) film. For co-network films with 60 to 90 wt% PSF (40:60, 25:75, and 10:90), sol content increased to ~16–20%, similarly to the PSF-only (0:100) film. The extracted sol was predominantly PSF as confirmed by TGA (Fig. S8).

### 3.3. Film thermal and shape memory properties

**Fig. 2.**
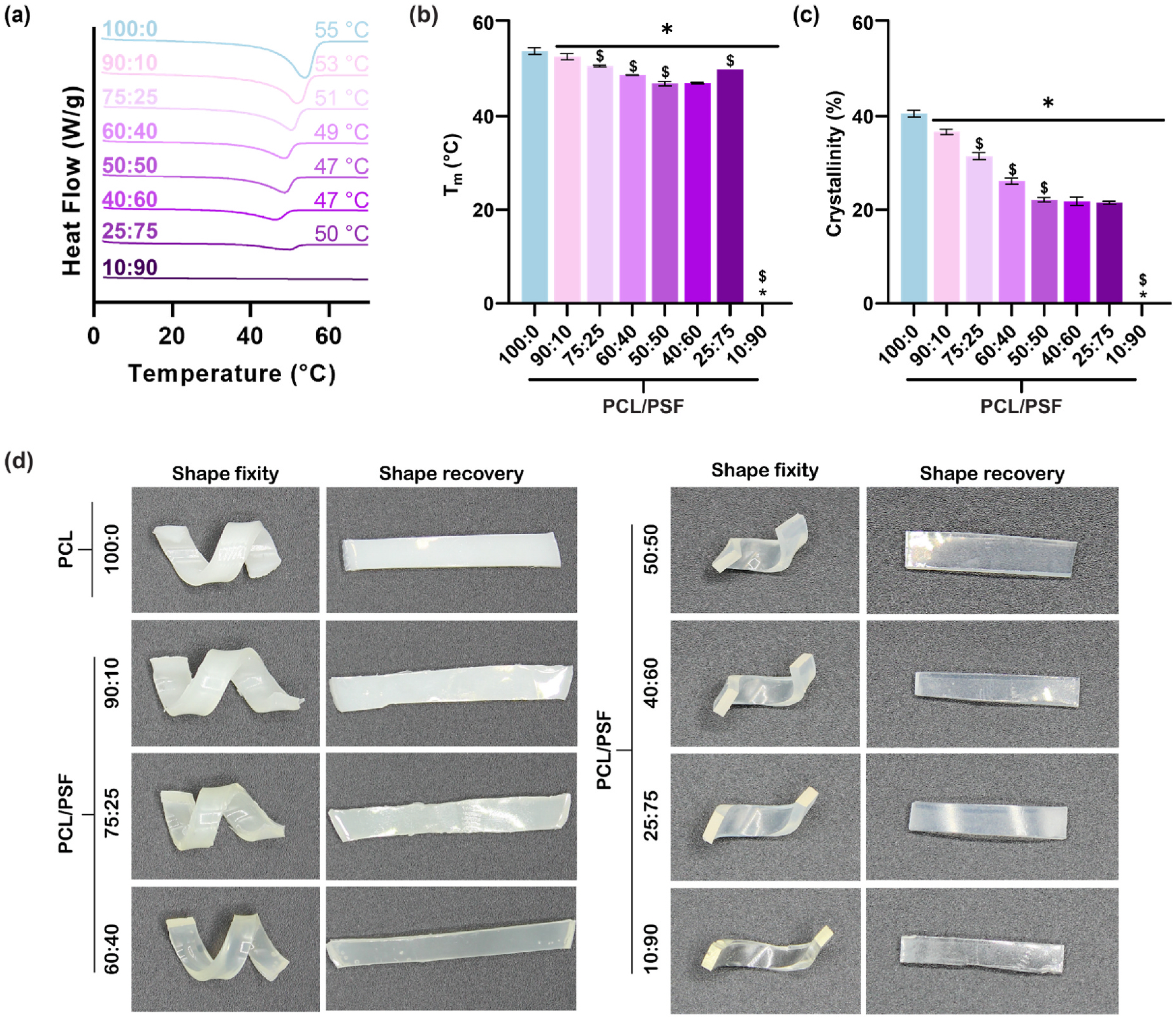
**(a)** Representative DSC thermograph of scaffolds. **(b)** *T*_*m, PCL*_ and **(c)** PCL % crystallinity values. *p < 0.05 vs PCL-only (100:0) control. $ *p* < 0.05 vs previous composition. **(d)** Photo series of films after shape fixation and subsequent shape recovery.

Given the importance to properties (e.g., mechanical, shape memory, and degradation), the *T*_*m, PCL*_ and % crystallinity of PCL of the PCL/PSF films were evaluated via DSC (Fig. 2a–c, Table S4). The PCL-only control (100:0) exhibited a *T*_*m, PCL*_ (~54 °C) and % crystallinity (~41%). For PCL/PSF films, the

*T*_*m, PCL*_ did not vary appreciably (~47–53 °C), but % crystallinity (~21–36%) decreased with increasing PSF content. For the 10:90 film (i.e., 90 wt% PSF), the *T*_*m, PCL*_ was completely lacking. Since PCL lamellae serve as switching segments, sufficient PCL crystallinity is necessary for shape memory behavior. Initially, a qualitative shape memory test was performed on films (Fig. 2d). As expected, due to high % PCL crystallinity, the PCL-only (100:0) control exhibited excellent shape fixity and recovery. This was likewise observed for PCL/PSF films with up to 40% PSF (90:10, 75:25, 60:40) and corresponding PCL crystallinity values of ~26 to ~37%. However, owing to an apparent insufficient PCL crystallinity (~22%), PCL/PSF films with ≥ 50% PSF (50:50, 40:60, 25:75, and 10:90) showed notable and progressive diminished shape memory behavior.

### 3.4. Scaffold fabrication and porosity

**Fig. 3.**
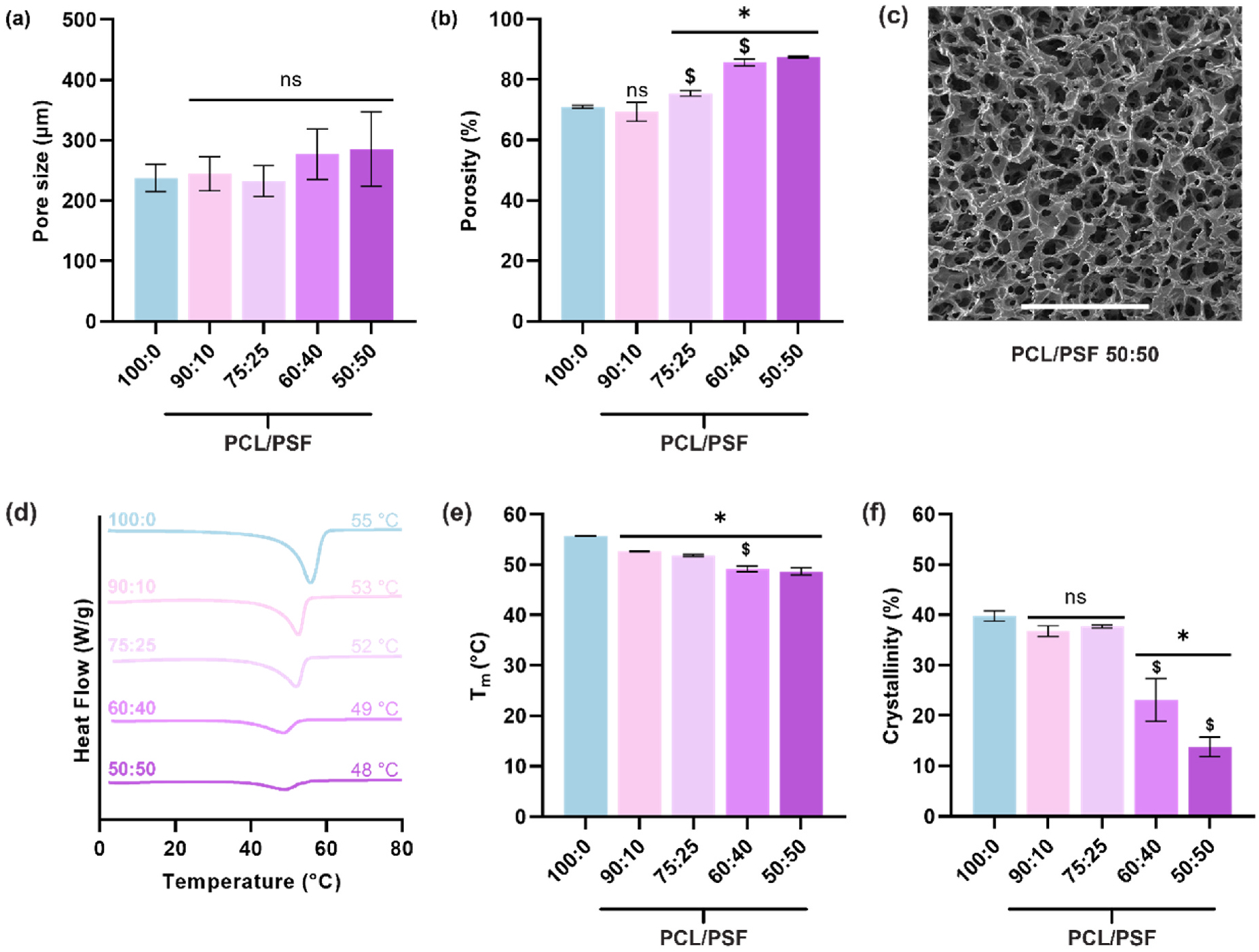
**(a)** Pore size and **(b)** % porosity values of scaffolds fabricated with adjusted salt size. **(c)** SEM image of PCL/PSF 50:50; scale bar = 1 mm. **(d)** DSC thermograms of scaffolds. **(e)** *T*_*m, PCL*_ values of scaffolds. **(f)** % PCL crystallinity values of scaffolds. *p < 0.05 vs PCL control. $ *p* < 0.05 vs previous composition noted in bar graph.

Based on the excellent crosslinking (< ~7% sol content) and shape memory behavior of films, PCL/PSF co-network scaffolds were prepared with up to 50 wt% PSF (90:10, 75:25, 60:40, and 50:50). The SCPL fabrication process employed a fused salt template to create interconnected macropores beneficial to cellular and tissue infiltration. Initially, scaffolds were fabricated from salt templates prepared with a sieved salt size of ~449 μm, like that used to prepare the PCL-only (100:0) scaffold. As for films, TGA thermograms of scaffolds reflected increased levels of PSF (Fig. S6c). Low sol content values (< ~8%) likewise verified that co-networks were successfully formed (Table S3). SEM images revealed that the average pore size of scaffolds increased with increasing PSF content, ranging from ~238 μm (100:0) to ~451 μm (50:50) (Table S5, Fig. S9a). This was attributed to reduced scaffold shrinkage during the final annealing step of scaffold fabrication. As reported previously, annealing is essential for shape memory behavior of PCL scaffolds, owing to reorganization of PCL lamellae ‘switching segments’ in closer proximity [14]. For co-network scaffolds prepared with PCL_90_-DA and siloxane macromers PDMS_66_-DMA or PMHS_66_-DMA (90:10, 75:25, and 60:40), there was no change in the extent of scaffold shrinkage upon annealing [16]. The reduced shrinkage of PCL/PSF scaffolds with increased PSF content may stem from the distinct ‘internal’ (i.e., in-chain) crosslinking of PSF versus the telechelic crosslinking of PDMS_66_-DMA or PMHS_66_-DMA. While all three siloxane macromers possess low *T*_*g*_s < 25 °C (*T*_*g, PSF*_ ~ −67 °C, *T*_*g, PDMS*_ ~ −120 °C, *T*_*g, PMHS*_ ~ −135 °C), the internal crosslinking of PSF may limit chain mobility and hence the reorganization of the PCL crystalline domains within the PCL/PSF co-networks. As PSF content increases, this effect is apparently more pronounced and leads to less annealing-induced scaffold shrinkage. A scaffold pore size of ~200–300 μm is desirable for bone regeneration [46,47], and a similar size would facilitate comparison of various compositions. Thus, the size of the sieved salt for the template was reduced accordingly for PCL/PSF scaffolds with greater PSF contents: 75:25 (~380 μm), 60:40 (~285 μm), and 50:50 (~285 μm). The resulting PCL/PSF scaffolds exhibited similar and desirable average pore sizes of ~250 μm, as well as high porosity (~71–87%) (Fig. 3a–c, Table S6, Fig. S9b). These scaffolds were subsequently utilized for evaluation of key material properties.

### 3.5. Scaffold T_m_, % crystallinity, and shape memory behavior

*T*_*m, PCL*_ is the transition temperature (i.e., *T*_*trans*_) required for shape actuation of the PCL and PCL/PSF scaffolds. It also represents the ‘fitting temperature’ (i.e., *T*_*fit*_) or the minimum exposure temperature required for self-fitting within the irregular bone defect. In prior studies, the PCL-only (100:0) scaffold was implanted into bone defects after submersion in 55 °C saline [9,10]. A lack of thermal necrosis was confirmed via histology, attributed to the rapid cooling of the scaffold upon removal from the warm saline and avoidance of direct irrigation of the defect/scaffold with 55+ °C saline following implantation. Since the *T*_*m, PCL*_ is above body temperature (~37 °C), mechanical rigidity was restored to the shape fixed scaffold within the defect. Per DSC analysis of PCL/PSF co-network scaffolds (Fig. 3d), as also observed for films, the *T*_*m, PCL*_ (~49–53 °C) were only slightly reduced versus the PCL-only (100:0) scaffold (~56 °C) (Fig. 3e–Table S4). PCL % crystallinity was sensitive to PSF content. Having relatively lower PSF contents, 90:10 and 75:25 exhibited statistically similar PCL % crystallinity (~40%) to the PCL-only (100:0) scaffold (Fig. 3f–Table 1, Table S4). At higher PSF contents, scaffold PCL % crystallinity was substantially reduced: 60:40 (~23%) and 50:50 (~14%). In contrast, co-network scaffolds prepared with PCL_90_-DA and siloxane macromers PDMS_66_-DMA or PMHS_66_-DMA (90:10, 75:25, and 60:40) not only maintained a *T*_*m*_, _*PCL*_ of ~55 °C, but largely maintained PCL crystallinity [16]. As noted previously, versus these telechelic crosslinking macromers, the distinct internal crosslinking of PSF may reduce PCL crystallinity by limiting organization of PCL segments into lamellae. Indeed, the reduction of PCL crystallinity of PCL/PSF 60:40 and 50:50 scaffolds coincide with aforementioned reduced annealing-induced shrinkage associated with reorganization of PCL crystalline lamellae. Despite the reduction in PCL crystallinity for these PCL/PSF scaffolds, they also displayed excellent shape fixity and shape recovery (> 92%) (Table 1, Table S7).

### 3.6. Mechanical properties

**Fig. 4.**
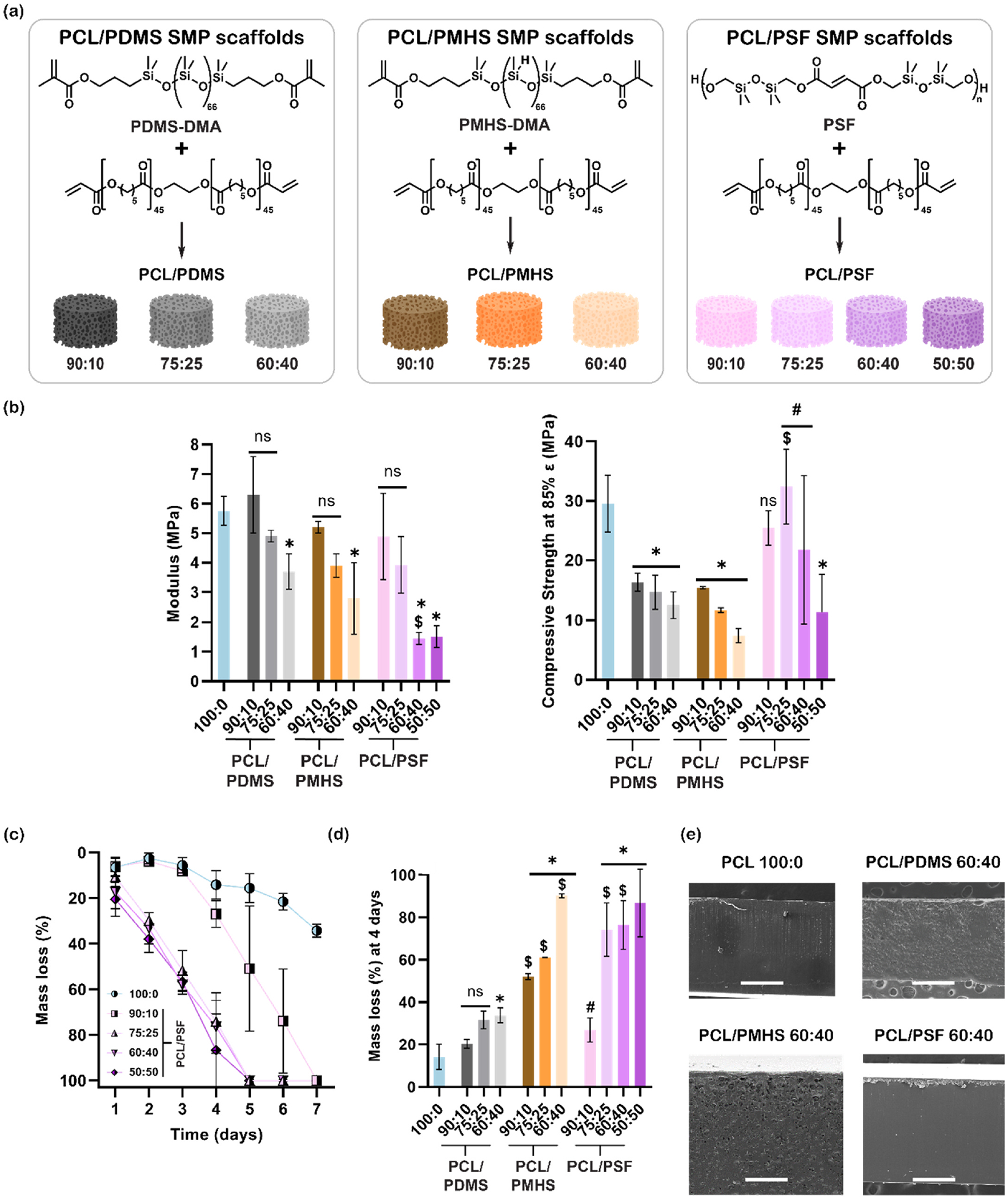
**(a)** Self-fitting SMP PCL/siloxane scaffolds prepared with varying wt% ratios of PCL_90_-DA to siloxane macromer (i.e., PDMS-DMA, PMHS-DMA, or PSF). **(b)** Scaffold compressive modulus (*E*) and strength (*CS*) (at 85% strain). **(c)** Based-catalyzed (0.2 M, 37 °C) degradation studies of PCL/PSF scaffolds, and **(d)** corresponding mass loss of scaffolds at 4 days for all compositions. **(e)** SEM images of neat (‘as prepared’) analogous films at 60:40 wt% PCL_90_-DA to siloxane macromer; scale bars = 500 μm *p < 0.05 vs PCL control (100:0). $ *p* < 0.05 vs analogous PDMS scaffolds. # *p* < 0.05 vs analogous PMHS scaffold. PCL/PDMS and PCL/PMHS data reported in Beltran et al. [16].

The mechanical properties of a scaffold intended to treat confined CMF bone defects are essential to its utility. The PCL-only (100:0) scaffold is mechanically robust, providing a compressive modulus (*E*) in the range of trabecular bone. Unlike conventional bone putties and cements, this PCL scaffold is also non-brittle as evident by its ability to withstand 85% strain, and thereby capable of promoting resistance to post-surgical fracture. These mechanical properties of the PCL-only scaffold contributed to its demonstrated capacity to support healing of a confined cranial defect [10] as well as a load-bearing femoral condyle defect [9]. Thus, for PCL/PSF co-network scaffolds, the optimal PSF content should be that which overall maintains these mechanical properties. In terms of rigidity, PCL/PSF scaffolds with relatively low PSF content (90:10 and 75:25) maintained statistically similar *E* values versus that of the PCL-only (100:0) scaffold (~6 MPa) (Fig. 4a, Table S8). This is attributed to their retention of PCL crystallinity levels (~40%). Additionally, the expected decrease in scaffold *E* with the inclusion of low *T*_*g*_ PSF (*T*_*g, PSF*_ ~ −67 °C) may be somewhat mitigated at low concentrations owing to its internal crosslinking to form the co-network PCL/PSF scaffolds. *E* values were also similar to those of PCL/PDMS (90:10 and 75:25) and PCL/PMHS (90:10 and 75:25) scaffolds at corresponding siloxane macromer contents. However, at higher PSF levels (60:40 and 50:50), scaffold *E* values were substantially reduced to ~1.5 MPa, attributed to the notable decrease in PCL crystallinity (~23 and ~14%) as well as the low PSF *T*_*g*_. Still, these *E* values remain appreciably higher than conventional hydrogel scaffolds (e.g., poly(ethylene glycol)-diacrylate, *M*_*n*_ ~3.4 kg mol^−1^, *E* ~3.4 kPa) [48–50]. While analogous PCL/PDMS (60:40) and PCL/PMHS (60:40) scaffolds also exhibited decreased *E* values (3–4 MPa) versus the PCL-only (100:0) scaffold, they were more rigid than PCL/PSF (60:40) owing to their retention of PCL % crystallinity. In terms of compressive strength (*CS*) (measured at 85% strain), only the PCL/PSF scaffold with the highest PSF content (50:50) (*CS* ~11 MPa) showed a reduction versus the PCL-only (100:0) scaffold (*CS* ~30 MPa) (Fig. 4b–Table S8). In contrast, PCL/PDMS and PCL/PMHS scaffolds showed reductions in *CS*, even at lowest siloxane macromer levels. Overall, PCL/PSF with 10 and 25 wt% PSF content (90:10 and 75:25) maintained the *E* and exceeded the *CS* of the PCL-only (100:0) scaffold. Additionally, these scaffolds maintained non-brittleness with no fracture at 85% strain.

### 3.7. Degradation behavior and bioactivity

Owing to their hydrophobicity and crystallinity, PCL is known to undergo prolonged (~2 years) degradation *in vivo* [11,12]. In contrast, bone regeneration rates are in the range of 4 to 12 weeks [51,52]. Thus, the PCL-only (100:0) scaffold’s expected slow resorption may limit neotissue infiltration. Modification of PCL-based SMP scaffolds to retain self-fitting and mechanical robustness while accelerating degradation is expected to improve bone tissue regeneration. Scaffold degradation was evaluated *in vitro* (0.2 M NaOH, 37 °C) per ASTM F1635 (Fig. 4c–Table S9, Fig. S10). PCL/PSF scaffolds degradation rates were dependent on PSF content. With just 10 wt% PSF, the PCL/PSF (90:10) scaffold degraded faster versus the PCL-only (100:0) scaffold. When PSF was increased to 25 wt%, the PCL/PSF (75:25) scaffold degraded appreciably faster. Under these conditions, similar degradation rates were observed for PCL/PSF scaffolds with higher PSF contents of 40 (60:40) and 50 wt% (50:50). The degradation rates of PCL/PSF scaffolds were different versus analogous PCL/PDMS and PCL/PMHS scaffolds, highlighted by comparing mass loss (t = 4 days) (Fig. 4d–Table S10). At the lowest siloxane macromer content (10 wt%), the PCL/PMHS (90:10) exhibited faster degradation. However, at 25 wt% siloxane macromer, PCL/PSF (75:25) exhibited a similar degradation rate versus analogous PCL/PMHS. These results reveal key differences contributing factors. Previous work showed that degradation of PCL-based systems can be accelerated via phase separation effects that afford greater water uptake and thus promote bond hydrolysis [13]. Phase separation was observed for PCL/siloxane co-network films prepared with PDMS_66_-DMA and PMHS_66_-DMA macromers [16] (Fig. 4e), resulting in faster degradation versus the PCL-only (100:0) scaffold (Fig. 4d). Thus, the accelerated degradation of PCL/PDMS and PCL/PMHS scaffolds is predominantly physically driven via phase separation, as these scaffolds retain PCL crystallinity and the PDMS and PMHS backbones are non-degradable, except for the telechelic ester crosslinks. PCL/PMHS scaffolds degrade faster versus PCL/PDMS scaffolds due to the relatively greater hydrophilicity of PMHS. Overall, since bulk erosion that accompanies polyester degradation is inherently heterogeneous [53], such phase separation may manifest the associated exponential loss in mechanical integrity [54]. In contrast, PCL/PSF scaffolds do not exhibit phase separation, even at high PSF levels (Fig. 4e, Fig. S11). The distinct inter-chain crosslinking of the PSF with PCL_90_-DA may have a compatibilizing effect. Accelerated degradation is particularly notable for PCL/PSF (75:25) since it occurs despite a lack of reduction in PCL crystallinity relative to the PCL-only (100:0) scaffold. These observations point to chemically-dominated accelerated degradation mechanism owing to the hydrolytically unstable PSF backbone. Thus, PCL/PSF scaffolds achieve accelerated degradation in the absence of phase separation and may potentially provide more homogeneous degradation.

In terms of bioactivity, PCL/PSF scaffolds did not exhibit HAp mineralization upon exposure to SBF (1X) after 30 days (Fig. S12). This is in contrast to PCL/PDMS (60:40) and PCL/PMHS (60:40) scaffolds that underwent HAp mineralization (SBF 1X) after 4 and 2 weeks, respectively [15,16]. It is hypothesized that this is due to the lower siloxane content of PSF. However, bioactivity, as well as further enhanced degradation rates, may be conferred to PCL/PSF scaffolds via the addition of a bioactive filler, as previously demonstrated for the PCL-only (100:0) and PCL/PDMS (75:25) scaffolds [55,56].

## 4. Conclusion

By providing conformal self-fitting, shape memory PCL scaffolds represent a promising alternative to treat CMF defects. Originally prepared from PCL_90_-DA via SCPL with a fused salt template, the resulting macroporous scaffolds achieved non-brittleness and rigidity (E ~6 MPa) in the range of trabecular bone. However, the slow degradation of PCL is expected to restrict neotissue infiltration. PCL/PDMS and PCL/PMHS scaffolds were subsequently formed by introducing telechelic siloxane macromers PDMS_66_-DMA and PMHS_66_-DMA, respectively. Degradation rates were increased stemming from phase separation effects, but remained limited by the siloxane macromers’ hydrolytically stable backbones. Herein, PSF was synthesized as a hybrid siloxane macromer with a hydrolytically unstable backbone as well as interchain crosslinkability, and used to prepare PCL/PSF co-network scaffolds. PCL/PSF films were initially prepared with varying wt% ratios of PCL to PSF (90:10, 75:25, 60:40, 50:50, 40:60, 25:75, and 10:90) to verify crosslinking and shape memory behavior. Based on these results, PCL/PSF scaffolds were prepared at 90:10, 75:25, 60:40, and 50:50 wt % ratios via SCPL. The PCL % crystallinity (~40%) was retained for scaffolds with relatively lower PSF content (90:10 and 75:25), but for those with higher PSF content was decreased to ~23% (60:40) and ~14% (50:50). This was attributed to PSF’s internal crosslinking that may limit PCL chain mobility and lamellae formation. This reduction in PCL crystallinity also resulted in reduced annealing-induced scaffold shrinkage during fabrication, prompting the use of smaller salt sizes for scaffolds with greater PSF content. Macroporous PCL/PSF scaffolds were obtained with a similar pore size (~250 μm) and with high porosities (~71–87%). Despite the reduction in PCL crystallinity for the aformentioned scaffolds, shape memory behavior was retained. Owing to their retention of PCL crystallinity, PCL/PSF 90:10 and 75:25 scaffolds maintain the *E* of the PCL-only (100:0) scaffold. *In vitro* degradation rates achieved by the PCL/PSF 75:25 scaffold notably surpassed that of analogous PCL/PDMS scaffold. A lack of phase separation was observed for PCL/PSF compositions, indicating that the hydrolytic instability of PSF contributes to accelerated degradation. Thus, PCL/PSF scaffolds with 25 wt% PSF afforded beneficial shape memory, mechanical, and degradation properties. Future studies of these PCL/PSF scaffolds will include evaluating the impact of sterilization (e.g., EtO and E-beam), *in vitro* cell culture (e.g., support of human MSC osteogenesis), and *in vivo* analysis in an appropriate bone defect model as described in our prior reports.

## Supplementary Material

1

## Figures and Tables

**Table 1:** Scaffold PCL % crystallinity, *T*_*m, PCL*_, shape fixity (*R*_*f*_) and recovery (*R*_*r*_) [*C* = 1].

	*T*_*m, PCL*_ (°C)	Crystallinity (%)	*R*_*f*_ (%)	*R*_*r*_ (%)
**PCL** **100:0**	55.7 ± 0.1	39.8 ± 1.0	95.0 ± 1.6	99.1 ± 1.5
**PCL/PSF** **90:10**	52.7 ± 0.1	36.8 ± 1.1	93.9 ± 0.8	100.0 ± 0.2
**PCL/PSF** **75:25**	51.9 ± 0.2	37.8 ± 0.3	93.0 ± 0.4	100.1 ± 0.8
**PCL/PSF** **60:40**	49.2 ± 0.6	23.1 ± 4.2	94.4 ± 1.8	98.6 ± 0.6
**PCL/PSF** **50:50**	48.7 ± 0.7	13.8 ± 1.9	95.0 ± 2.3	99.3 ± 0.6

## Data Availability

Statement Raw data is available via the Texas Data Repository (TDR).

## Appendix A. Supplementary data

Supplementary data to this article can be found online at https://doi.org/10.1016/j.polymer.2026.129694.
