# Supplementary material for: Biodegradable poly(ε-caprolactone)/poly(silyl fumarate) shape memory scaffolds
Source: Polymer (Guildf). Author manuscript; Available in PMC 2026 Jul 15. (PMC13367451; doi:10.1016/j.polymer.2026.129694)
Supplement: 1 [file NIHMS2191102-supplement-1.docx]

Supporting Information

**Biodegradable Poly(ε-caprolactone)/Poly(silyl fumarate)
Shape Memory Scaffolds**

Jenlyan Negrón Hernández^1^, Kaley Beach^2^, Paola Chavarria^3^, Melissa A Grunlan^1,2,3^

^1^Department of Chemistry, ^2^Department of Materials Science and Engineering, ^3^Department of Biomedical Engineering, Texas A&M University, College Station, Texas 77843, United States.

*Corresponding author email: [mgrunlan@tamu.edu](mailto:mgrunlan@tamu.edu)

18 Pages

12 Figures

10 Tables

**
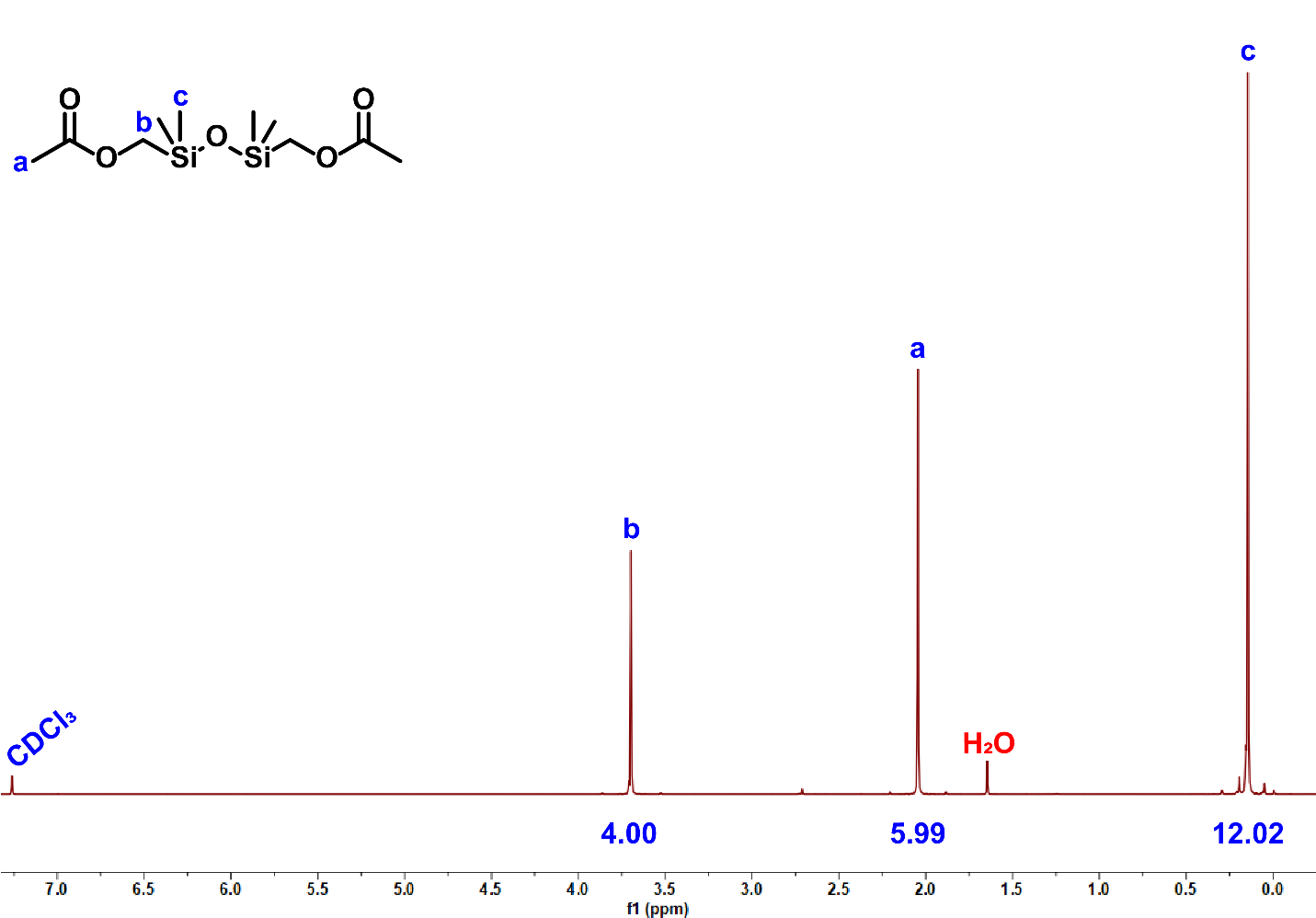
**

**Figure S1.** ^1^H-NMR of 1,3-Bis(acetoxymethyl)tetramethyldisiloxane **(a)** (CDCl_3_; δ, ppm): 0.10-0.18 (s, 12H, SiCH_3_), 1.99-2.08 (s, 6H, SiCH_2_O), 3.66-3.73 (s, O=CCH_3_).

**
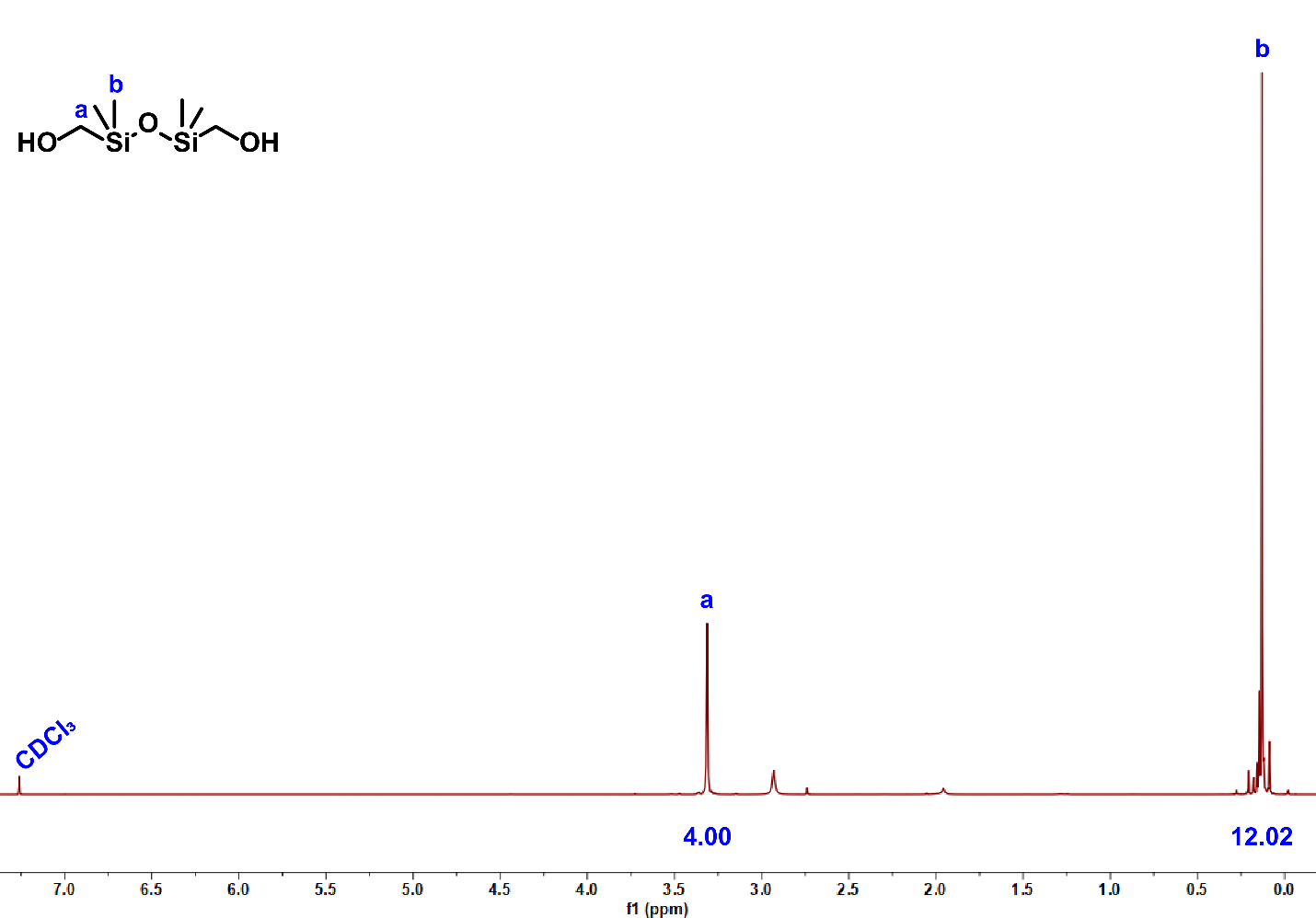
**

**Figure S2.** ^1^H-NMR of 1,3-Bis(hydroxymethyl)tetramethyldisiloxane **(b)** (CDCl_3_; δ, ppm): 0.10-0.18 (s, 12H, SiCH_3_), 3.21-3.40 (s, SiCH_2_O).

**
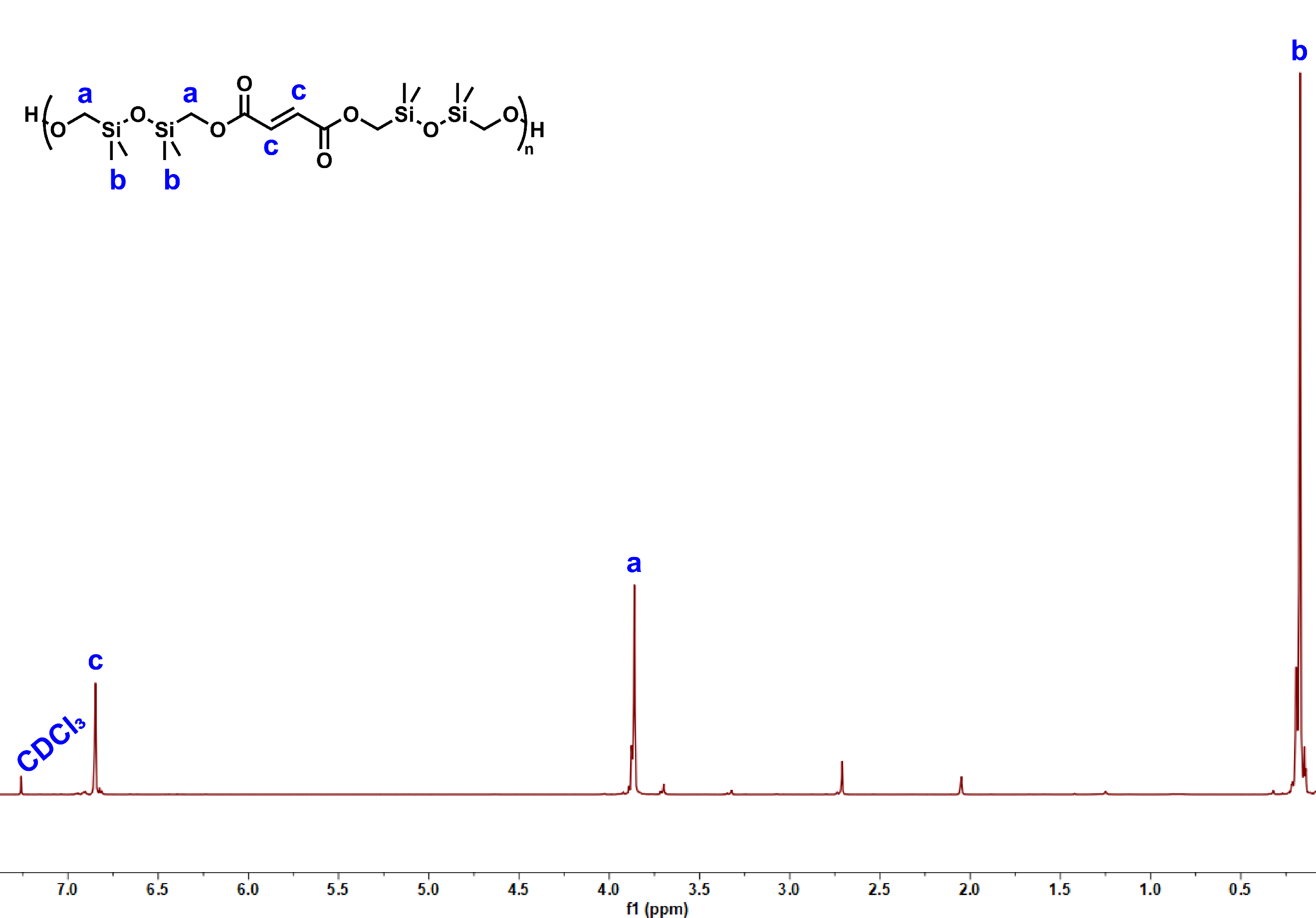
**

**Figure S3.** ^1^H-NMR of PSF (CDCl_3_; δ, ppm): 0.15-0.19 (s, 12H, SiCH_3_), 3.78-3.97 (4H, SiCH_2_O), 6.78-6.93 (s, 2H, CH=CH).

**Table S1:** SEC analysis of PSF.

| **Peak** | ***M_w_* (g mol^-1^)** | ***M_n_* (g mol^-1^)** | **Dispersity (Ð)** |
| --- | --- | --- | --- |
| **1** | 95,000 | 82,000 | 1.2 |
| **2** | 9000 | 5000 | 1.8 |
| **3** | 300 | 100 | 1.8 |

*Weighted *M_w_* of peaks 1-3 = ~16 kg mol^-1^, Ð ~28

**
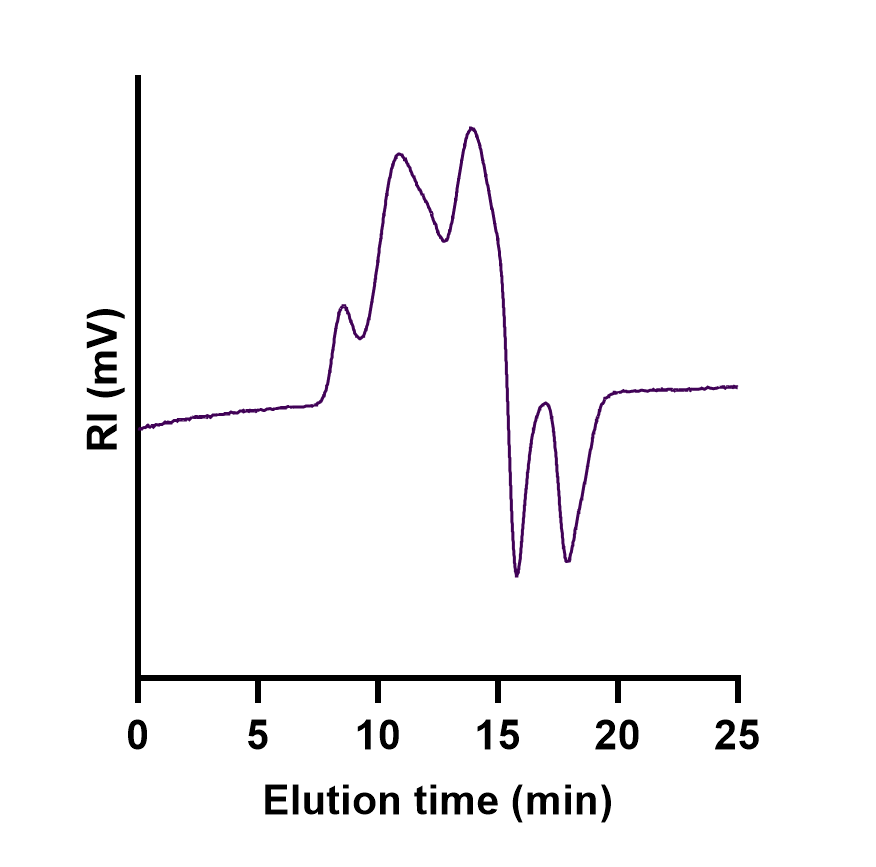
**

**Figure S4.** SEC trace of PSF.

**Table S2:** DSC analysis of PSF.

|  | ***T_g_* (°C)** |
| --- | --- |
| **PSF** | -67.3 ± 0.3 |


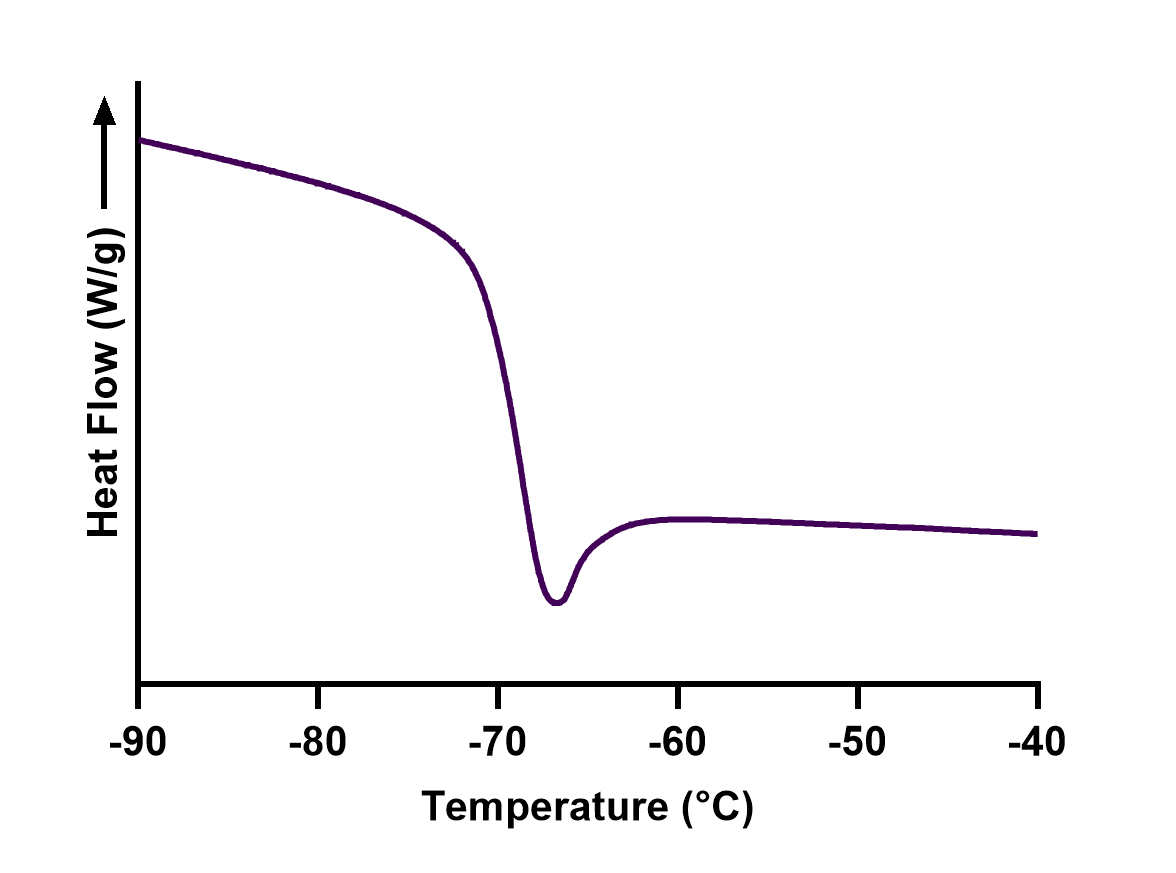


**Figure S5.** DSC thermogram of PSF.


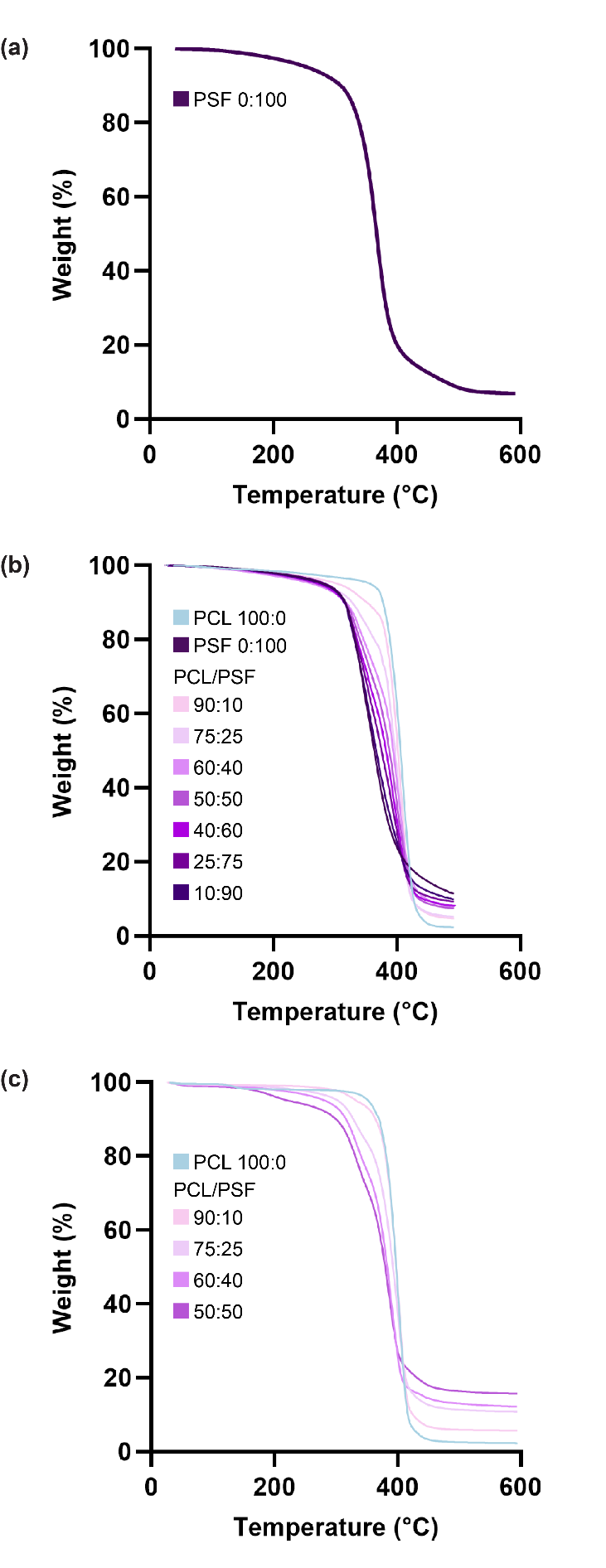


**Figure S6.** TGA of **(a)** PSF (copolymer), **(b)** PCL/PSF films, and **(c)** PCL/PSF scaffolds.

**
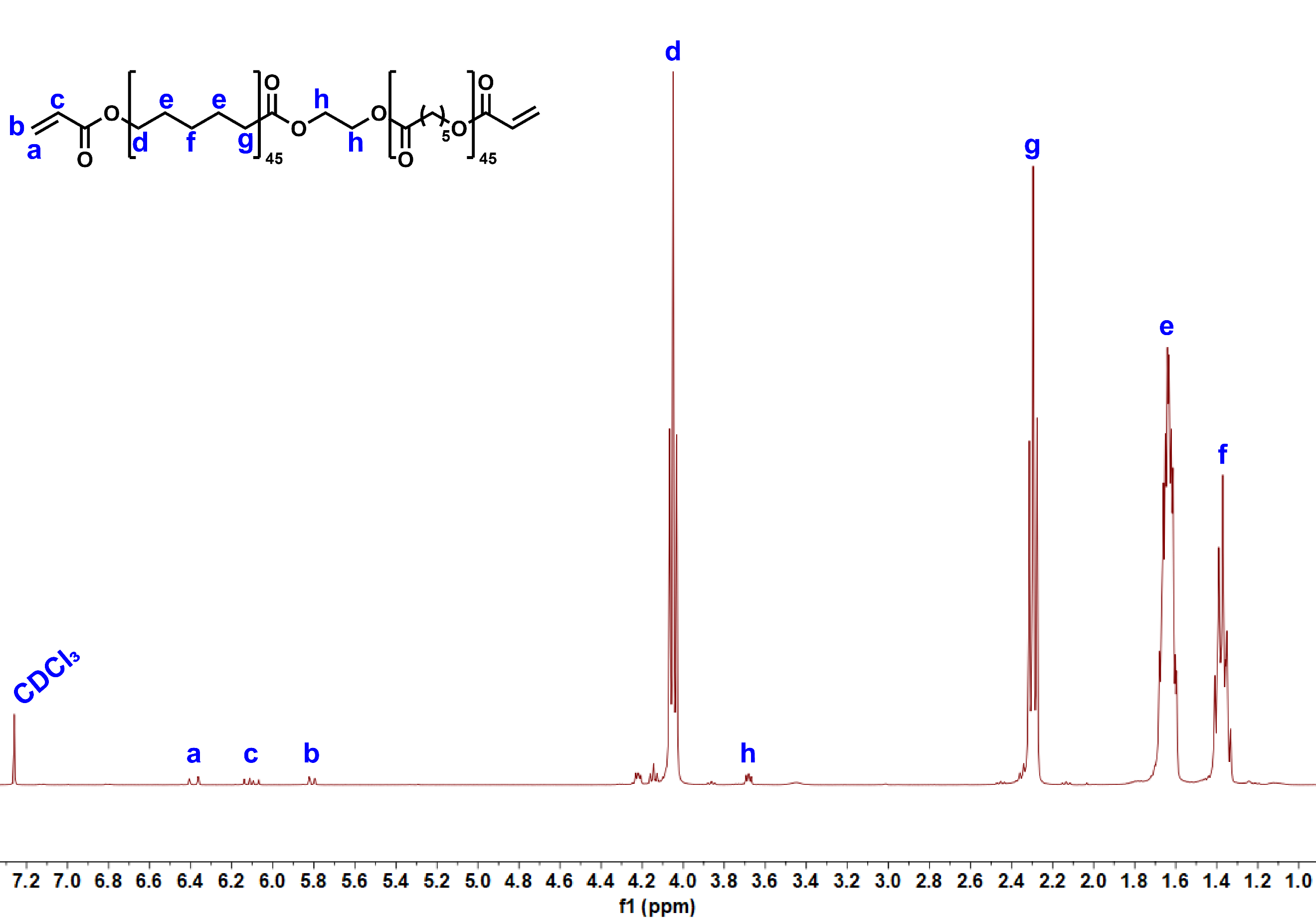
**

**Figure S7.** ^1^H-NMR of PCL_90_-DA (CDCl_3_; δ, ppm): 1.25-1.49 (m, 180H, CH_2_CH_2_CH_2_), 1.52-1.90 (m, 360H, CH_2_CH_2_CH_2_), 2.22-2.43 (t, 180H, CH_2_CH_2_C=O), 3.64-3.73 (t, 4H, OCH_2_CH_2_O), 3.93-4.32 (t, 180H, OCH_2_CH_2_, 5.71-5.87 (d, 2H, CH=CH=CH), 6.02-6.19 (m, 2H, OCCH=CH_2_), 6.32-6.44 (d, 2H, CH=CH=CH).

**Table S3:** Sol content of films and scaffolds.

|  | **Sol content (%)**  **Films** | **Sol content (%)**  **Scaffolds** |
| --- | --- | --- |
| **PCL**  **100:0** | 7.8 ± 0.1 | 5.8 ± 2.8 |
| **PCL/PSF**  **90:10** | 6.2 ± 0.6 | 3.4 ± 2.5 |
| **PCL/PSF**  **75:25** | 5.7 ± 1.0 | 5.8 ± 2.6 |
| **PCL/PSF**  **60:40** | 4.0 ± 1.1 | 7.8 ± 1.3 |
| **PCL/PSF**  **50:50** | 7.0 ± 1.2 | 4.5 ± 0.4 |
| **PCL/PSF**  **40:60** | 20.3 ± 10.7 | - |
| **PCL/PSF**  **25:75** | 21.1 ± 3.2 | - |
| **PCL/PSF**  **10:90** | 16.5 ± 2.33 | - |
| **PSF**  **0:100** | 23.9 ± 5.1 | - |


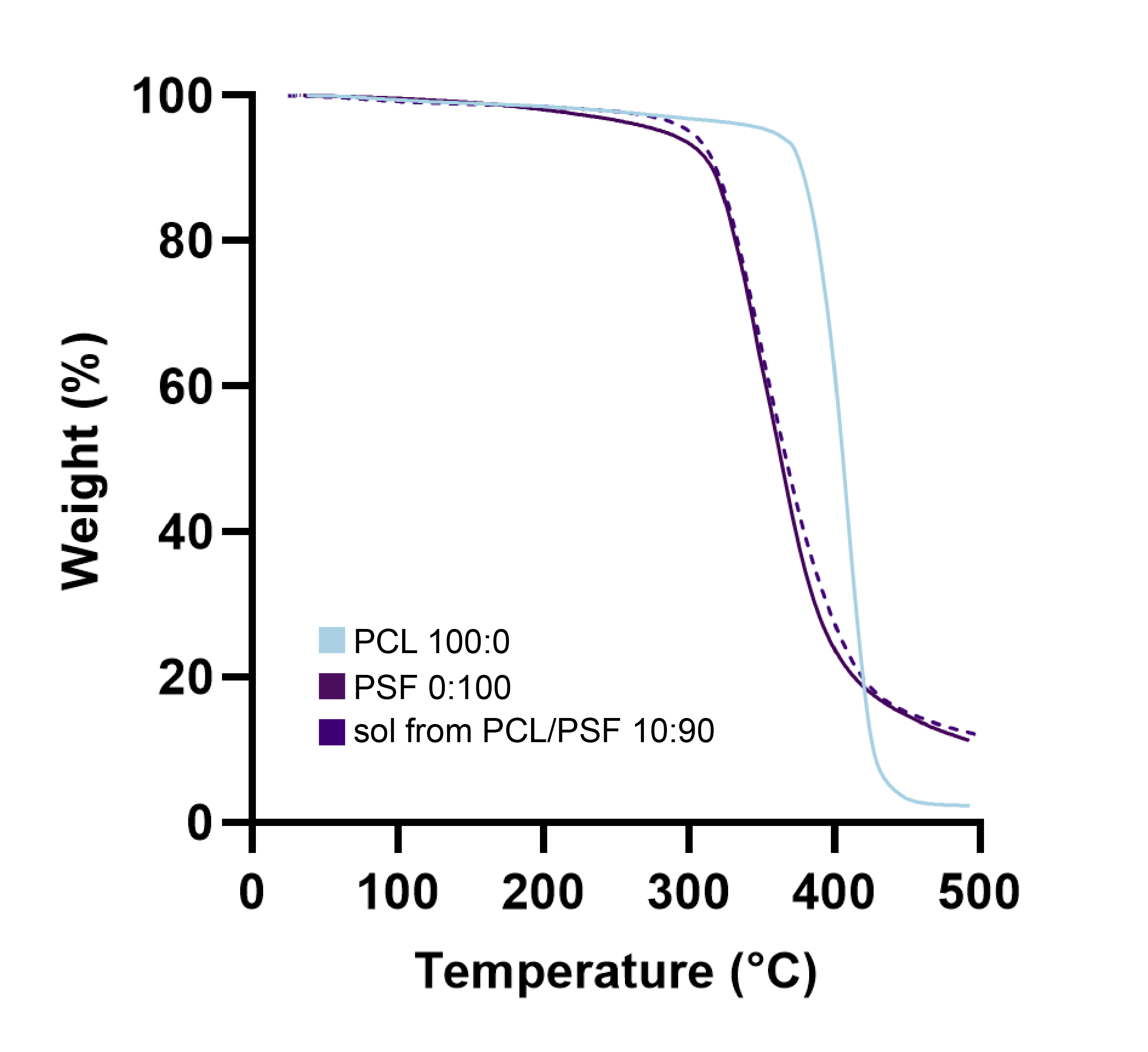


**Figure S8.** TGA of PCL-only (100:0) film, PSF-only (0:100) film, and extracted sol from PCL/PSF (10:90) films.

**Table S4:** Thermal properties of films and scaffolds.

|  | **Films** | | | **Scaffolds** | | |
| --- | --- | --- | --- | --- | --- | --- |
|  | | ***T_m, PCL_* midpoint  (°C)** | **Crystallinity (%)** | | ***T_m, PCL_* midpoint (°C)** | **Crystallinity (%)** |
| **PCL**  **100:0** | | 53.9 ± 0.7 | 40.6 ± 0.7 | | 55.7 ± 0.1 | 39.8 ± 1.0 |
| **PCL/PSF**  **90:10** | | 52.7 ± 0.5 | 36.7 ± 0.6 | | 52.7 ± 0.1 | 36.8 ± 1.1 |
| **PCL/PSF**  **75:25** | | 50.7 ± 0.2 | 31.5 ± 0.8 | | 51.9 ± 0.2 | 37.8 ± 0.3 |
| **PCL/PSF**  **60:40** | | 48.8 ± 0.4 | 26.2 ± 0.6 | | 49.2 ± 0.6 | 23.1 ± 4.2 |
| **PCL/PSF**  **50:50** | | 47.0 ± 0.5 | 22.2 ± 0.5 | | 48.7 ± 0.7 | 13.8 ± 1.9 |
| **PCL/PSF**  **40:60** | | 47.1 ± 0.2 | 21.8 ± 0.9 | | - | - |
| **PCL/PSF**  **25:75** | | 50.0 ± 0.0 | 21.5 ± 0.3 | | - | - |
| **PCL/PSF**  **10:90** | | - | - | | - | - |

**Table S5:** Scaffolds pore size and % porosity when prepared with templates of same sieve salt size (449 ± 36 µm).

|  | **Sieved salt (µm)** | **Porosity (%)** | **Pore size (µm)** |
| --- | --- | --- | --- |
| **PCL**  **100:0** | 449 ± 36 µm | 71.0 ± 0.4 | 238 ± 23 |
| **PCL/PSF**  **90:10** | 449 ± 36 µm | 69.3 ± 3.1 | 245 ± 28 |
| **PCL/PSF**  **75:25** | 449 ± 36 µm | 76.3 ± 3.1 | 355 ± 27 |
| **PCL/PSF**  **60:40** | 449 ± 36 µm | 81.8 ± 3.9 | 422 ± 38 |
| **PCL/PSF**  **50:50** | 449 ± 36 µm | 84.4 ± 0.9 | 451 ± 39 |


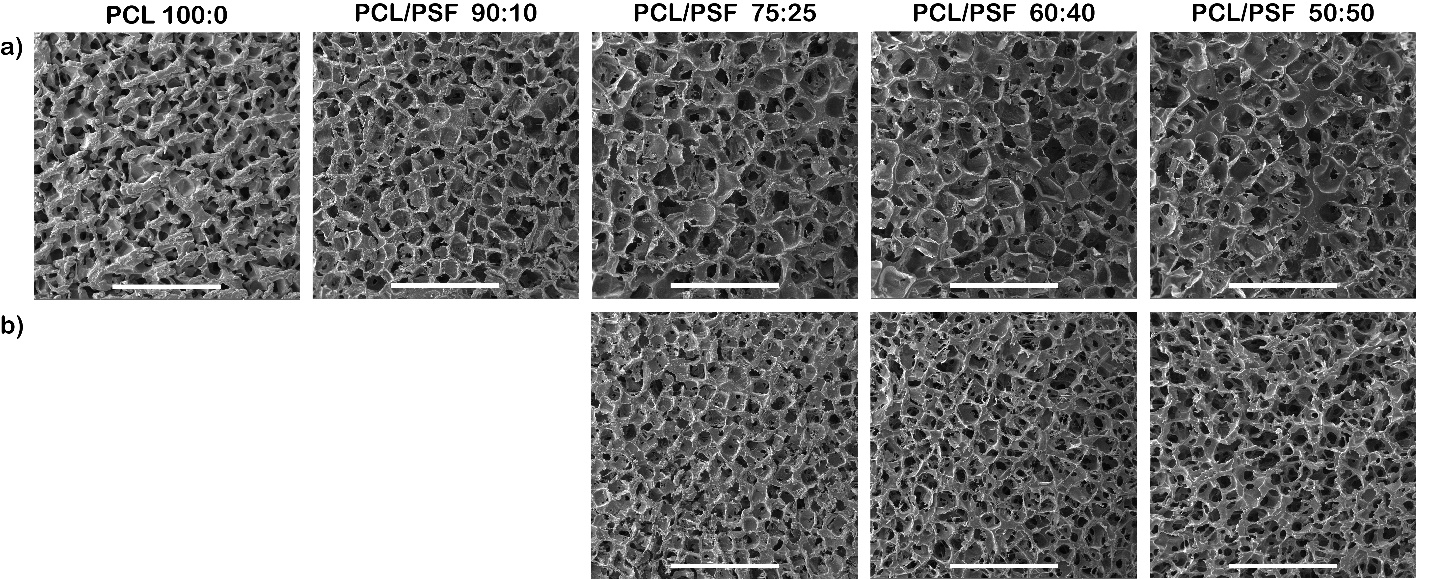


**Figure S9.** SEM images of PCL and PCL/PSF scaffold cross-sections. **(a)** Scaffolds prepared with same salt size (449 ± 36 µm), and **(b)** scaffolds prepared with different salt size: 75:25 (380 ± 58 µm), 60:40 (285 ± 70 µm), and 50:50 (285 ± 70 µm). Scale bars = 1 mm.

**Table S6:** Scaffold porosity and pore size when prepared with templates of varying sieved salt sizes. (Note: These scaffolds were used for subsequent analyses.)

|  | **Sieved salt (µm)** | **Porosity (%)** | **Pore size (µm)** |
| --- | --- | --- | --- |
| **PCL**  **100:0** | 449 ± 36 µm | 71.0 ± 0 | 238 ± 23 |
| **PCL/PSF**  **90:10** | 449 ± 36 µm | 69 ± 3 | 245 ± 28 |
| **PCL/PSF**  **75:25** | 380 ± 58 µm | 75 ± 1 | 232 ± 26 |
| **PCL/PSF**  **60:40** | 285 ± 70 µm | 86 ± 1 | 277 ± 42 |
| **PCL/PSF**  **50:50** | 285 ± 70 µm | 87 ± 0 | 286 ± 62 |

**Table S7:** Shape memory behavior of scaffolds: shape fixity shape fixity (*R_f_*) and recovery (*R_r_*) for cycle 0 [‘pre-cycle’] (*C* = 0) and subsequent cycle (*C* = 1).

|  | | ***C* = 0** | | | ***C* = 1** | |
| --- | --- | --- | --- | --- | --- | --- |
|  | ***R_f_* (%)** | | ***R_r_* (%)** | ***R_f_* (%)** | | ***R_r_* (%)** |
| **PCL**  **100:0** | 92.9 ± 2.0 | | 101.5 ± 1.2 | 95.0 ± 1.6 | | 99.1 ± 1.5 |
| **PCL/PSF**  **90:10** | 93.3 ± 1.8 | | 100.9 ± 0.8 | 93.9 ± 0.8 | | 100.0 ± 0.2 |
| **PCL/PSF**  **75:25** | 93.4 ± 0.4 | | 100.9 ± 0.4 | 93.0 ± 0.4 | | 100.1 ± 0.8 |
| **PCL/PSF**  **60:40** | 94.3 ± 2.3 | | 99.9 ± 0.8 | 94.4 ± 1.8 | | 98.6 ± 0.6 |
| **PCL/PSF**  **50:50** | 96.8 ± 1.0 | | 99.6 ± 0.5 | 95.0 ± 2.3 | | 99.3 ± 0.6 |

**Table S8:** Compressive mechanical properties of PCL, PCL/PDMS, PCL/PMHS, and PCL/PSF scaffolds.

|  | **Modulus (MPa)** | **Strength (MPa)** |
| --- | --- | --- |
| **PCL**  **100:0** | 5.8 ± 0.5 | 29.5 ± 4.8 |
| **PCL/PDMS***  **90:10** | 6.3 ± 1.3 | 16.3 ± 1.5 |
| **PCL/PDMS***  **75:25** | 4.9 ± 0.2 | 14.7 ± 2.8 |
| **PCL/PDMS***  **60:40** | 3.7 ± 0.6 | 12.5 ± 2.2 |
| **PCL/PMHS***  **90:10** | 5.2 ± 0.2 | 15.4 ± 1.3 |
| **PCL/ PMHS***  **75:25** | 3.9 ± 0.4 | 11.7 ± 1.8 |
| **PCL/ PMHS***  **60:40** | 2.8 ± 1.2 | 7.4 ± 3.1 |
| **PCL/PSF**  **90:10** | 4.9 ± 1.5 | 25.5 ± 2.9 |
| **PCL/PSF**  **75:25** | 3.9 ± 1.0 | 32.4 ± 6.3 |
| **PCL/PSF**  **60:40** | 1.5 ± 0.2 | 21.8 ± 12.4 |
| **PCL/PSF**  **50:50** | 1.5 ± 0.4 | 11.4 ± 6.3 |

*PCL/PDMS and PCL/PMHS data reported in F.O. Beltran, *et al*. [1]

**Table S9:** PCL and PCL/PSF scaffolds % mass loss (0.2 M NaOH, 37 °C).

|  | **1 d** | **2 d** | **3 d** | **4 d** | **5 d** | **6 d** | **7 d** |
| --- | --- | --- | --- | --- | --- | --- | --- |
| **PCL**  **100:0** | 6.6 ± 4.0 | 2.7 ± 2.4 | 5.6 ± 3.3 | 14.2 ± 6.0 | 15.7 ± 6.3 | 21.6 ± 3.6 | 34.2 ± 2.7 |
| **PCL/PSF**  **90:10** | 6.2 ± 4.1 | 3.9 ± 2.0 | 8.1 ± 2.1 | 26.9 ± 5.7 | 74.7 ± 29.2 | 74.0 ± 22.8 | 100.0 ± 0.0 |
| **PCL/PSF**  **75:25** | 10.7 ± 4.0 | 29.8 ± 1.6 | 51.8 ± 8.7 | 74.2 ± 12.6 | 100.0 ± 0.0 | 100.0 ± 0.0 | 100.0 ± 0.0 |
| **PCL/PSF**  **60:40** | 17.4 ± 7.2 | 33.2 ± 6.8 | 58.1 ± 4.0 | 76.5 ± 11.5 | 100.0 ± 0.0 | 100.0 ± 0.0 | 100.0 ± 0.0 |
| **PCL/PSF**  **50:50** | 20.6 ± 7.3 | 38.0 ± 5.8 | 56.7 ± 4.1 | 86.8 ± 15.9 | 100.0 ± 0.0 | 100.0 ± 0.0 | 100.0 ± 0.0 |


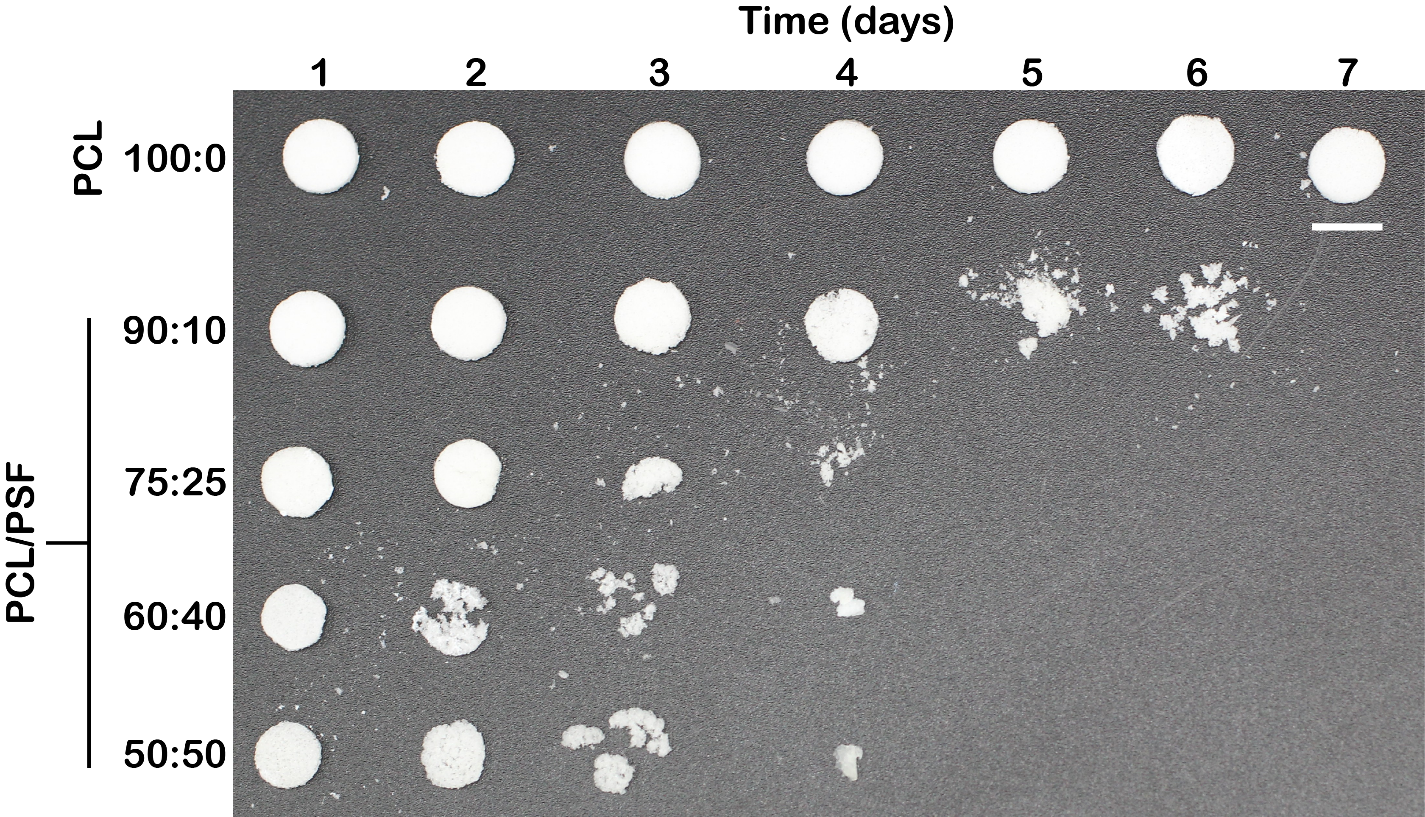


**Figure S10.** Photo-series of PCL and PCL/PSF scaffolds during accelerated degradation study. Scale bar = 6 mm.

**Table S10:** PCL, PCL/PDMS, PCL/PMHS, and PCL/PSF scaffolds mass loss (%) at 4 days (0.2 M NaOH, 37 °C).

|  | **Mass loss (%)** |
| --- | --- |
| **PCL**  **100:0** | 14.2 ± 6.0 |
| **PCL/PDMS***  **90:10** | 20.3 ± 2.1 |
| **PCL/ PDMS***  **75:25** | 31.7 ± 4.2 |
| **PCL/ PDMS***  **60:40** | 33.9 ± 3.5 |
| **PCL/PMHS***  **90:10** | 52.1 ± 1.4 |
| **PCL/ PMHS***  **75:25** | 61.1 ± 0.1 |
| **PCL/ PMHS***  **60:40** | 90.2 ± 0.9 |
| **PCL/PSF**  **90:10** | 26.9 ± 5.7 |
| **PCL/PSF**  **75:25** | 74.2 ± 12.6 |
| **PCL/PSF**  **60:40** | 76.5 ± 11.5 |
| **PCL/PSF**  **50:50** | 86.8 ± 15.9 |

*PCL/PDMS and PCL/PMHS data reported in F.O. Beltran, *et al*. [1]

**
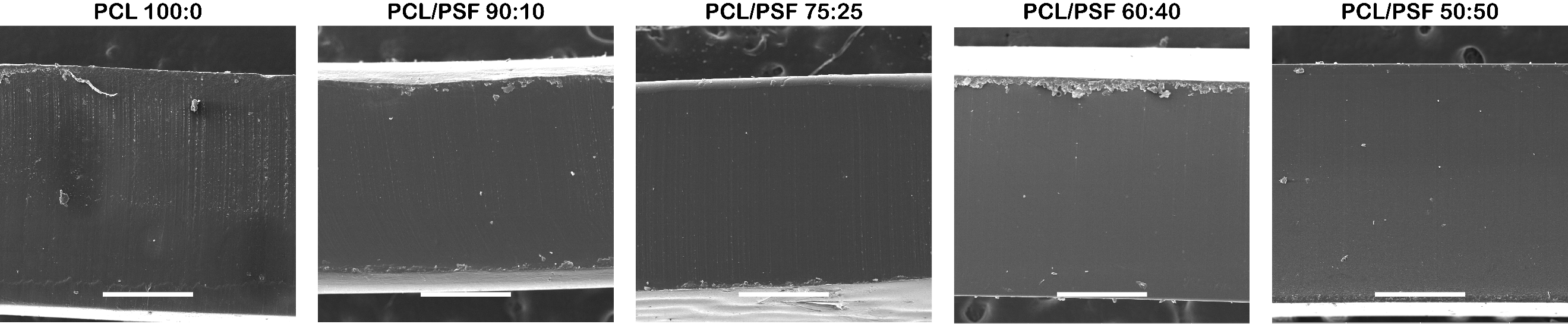
**

**Figure S11.** SEM images of PCL and PCL/PSF film cross-sections. Scale bars = 500 µm.

**References**


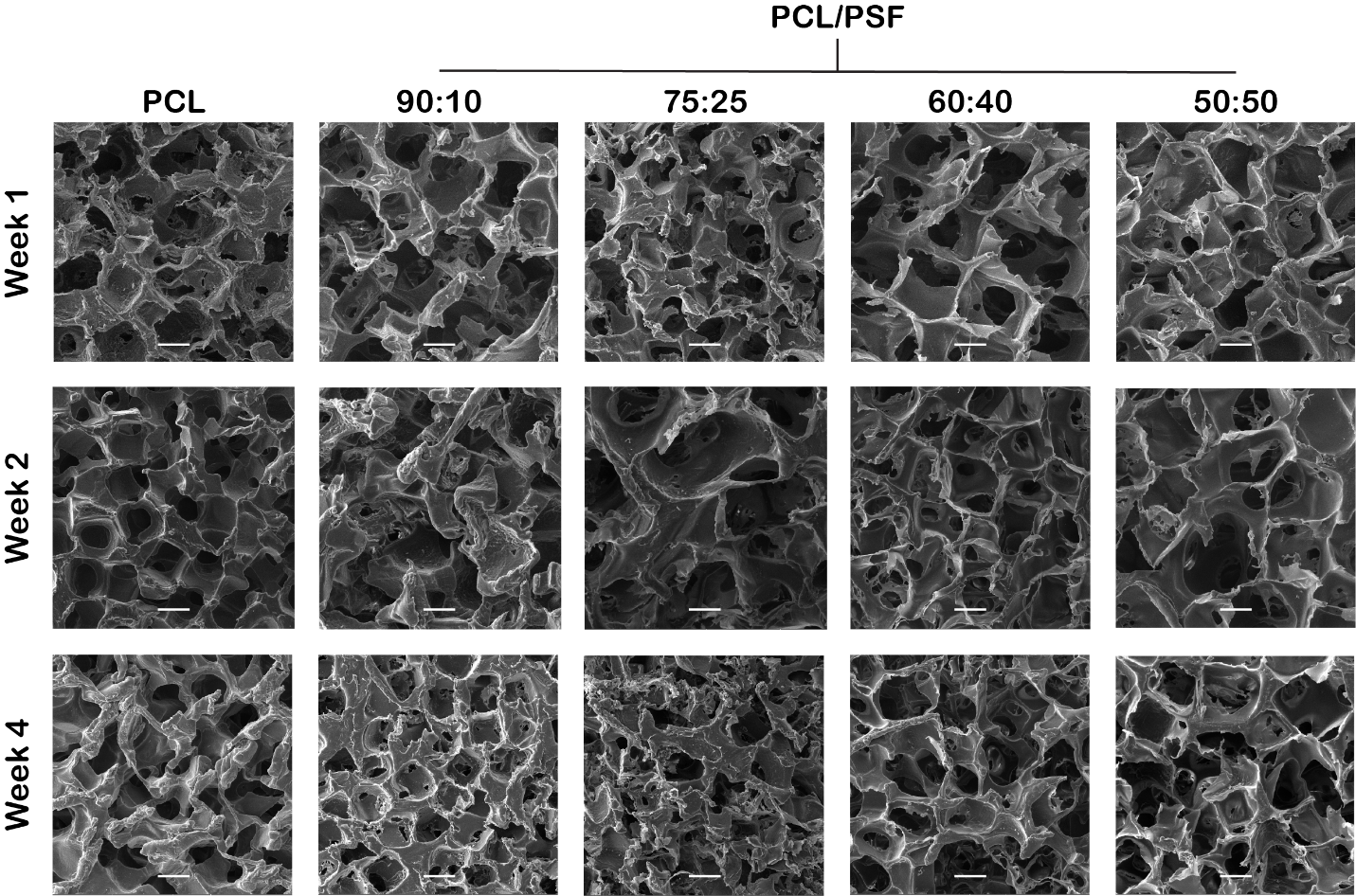


**Figure S12.** SEM images of scaffolds cross-section after exposure to 1X SBF. Scale bars = 200 µm.

[1] F.O. Beltran, A.S. Arabiyat, R.A. Culibrk, D.J. Yeisley, C.J. Houk, A.J. Hicks, J. Negrón Hernández, B.M. Nitschke, M.S. Hahn, M.A. Grunlan, Enhanced degradation and bioactivity in polysiloxane-based shape memory polymer (SMP) scaffolds, Polymer 284 (2023) 126291.
